# Targeted protein degradation dismantles undruggable targets to reverse immune evasion and therapy resistance in cancer

**DOI:** 10.3389/fimmu.2026.1935909

**Published:** 2026-09-08

**Authors:** Furong Zhang, Qianheng Wang, Mingxuan Li, Tingting Wang, Ping Lei, Tao Huang, Zhenghao Wu

**Affiliations:** 1 Department of Breast and Thyroid Surgery, Union Hospital, Tongji Medical College, Huazhong University of Science and Technology, Wuhan, China; 2 Department of Immunology, School of Basic Medicine, Tongji Medical College, Huazhong University of Science and Technology, Wuhan, China; 3 Guangdong Provincial Key Laboratory of Malignant Tumour Epigenetics and Gene Regulation, Guangdong-Hong Kong Joint Laboratory for ribonucleic acid (RNA) Medicine, Breast Tumour Center, Sun Yat-sen Memorial Hospital, Sun Yat-sen University, Guangzhou, China

**Keywords:** cancer, immune evasion, targeted protein degradation, therapy resistance, undruggable targets

## Abstract

Targeted protein degradation (TPD) harnesses endogenous proteolytic machineries—the ubiquitin–proteasome system and lysosomal pathways—to selectively eliminate disease-causing proteins that are refractory to conventional inhibition. In cancer immunotherapy, TPD dismantles critical immunosuppressive nodes across extracellular, membrane and intracellular compartments, reprogramming the tumor microenvironment from an immunologically ‘cold’ to a ‘hot’ state. This Review examines the molecular engineering principles of bifunctional degraders and summarizes clinical progress demonstrating synergy with immune checkpoint blockade and adoptive cell therapy. We discuss how catalytic protein elimination overcomes primary immune evasion and adaptive resistance driven by compensatory signaling, target mutation and metabolic rewiring. Finally, we outline translational roadblocks in cell-specific delivery, therapeutic window optimization and on-target/off-tissue toxicity mitigation, and propose engineering strategies to advance the clinical implementation of targeted protein degradation in immuno-oncology.

## 1 Introduction

Originating from the conceptualization of proteolysis-targeting chimeras (PROTAC) two decades ago (1), targeted protein degradation (TPD) has emerged as an emerging therapeutic modality that catalyzes the physical elimination—rather than transient inhibition—of disease-causing proteins (2). By co-opting endogenous proteolytic machineries — the ubiquitin–proteasome system (UPS) or lysosome-targeting receptor (LTR) —TPD expands the druggable target space to encompass “undruggable” immunomodulatory proteins, including transcription factors, scaffolding proteins and extracellular cytokines that drive tumor immune evasion (3).

Over the past decade, TPD has transitioned from chemical biology curiosities to clinically validated therapeutics (4). KT-333, a Signal Transducer and Activator of Transcription 3 (STAT3) degrader, has demonstrated complete remissions in refractory lymphomas. Together with the entry of vepdegestrant (ARV-471) into Phase III trials for breast cancer, these findings establish that catalytic protein elimination can overcome resistance to conventional immunotherapies (5, 6). Concurrently, preclinical advances in lysosome-targeting chimeras (LYTACs) and antibody-based degraders (AbTACs) have extended this approach to membrane checkpoints and secreted immunosuppressive factors (7, 8).

However, the clinical translation of TPD for immuno-oncology remains confronted with substantial technical and biological hurdles. While TPD offers a mechanistic solution to resistance, challenges in cell-specific delivery, optimization of the therapeutic window (including the hook effect), and mitigation of on-target/off-tissue toxicities constrain its current clinical impact (9, 10).

Here we summarize translational advances in TPD for cancer immunotherapy, organizing discussion by target localization—membrane, extracellular and intracellular compartments. We emphasize clinical evidence demonstrating efficacy against resistant disease, and outline strategies to optimize cell-specific delivery, therapeutic windows and safety profiles.

The rationale for applying TPD to cancer immunotherapy rests on three observations: (i) many immuno-oncology targets (e.g., STAT3, FOXP3) lack druggable pockets or possess protein–protein interaction interfaces intractable to competitive inhibition; (ii) conventional inhibitors frequently fail due to adaptive resistance mechanisms including target mutation, compensatory pathway upregulation, and stromal barriers; and (iii) TPD’s catalytic pharmacology—one degrader molecule eliminating multiple target copies—offers sustained target suppression at sub-stoichiometric concentrations, a feature particularly attractive for overcoming high-burden or high-turnover immunosuppressive proteins.

## 2 TPD technology

### 2.1 Concept and fundamental principles

**Figure 1:**
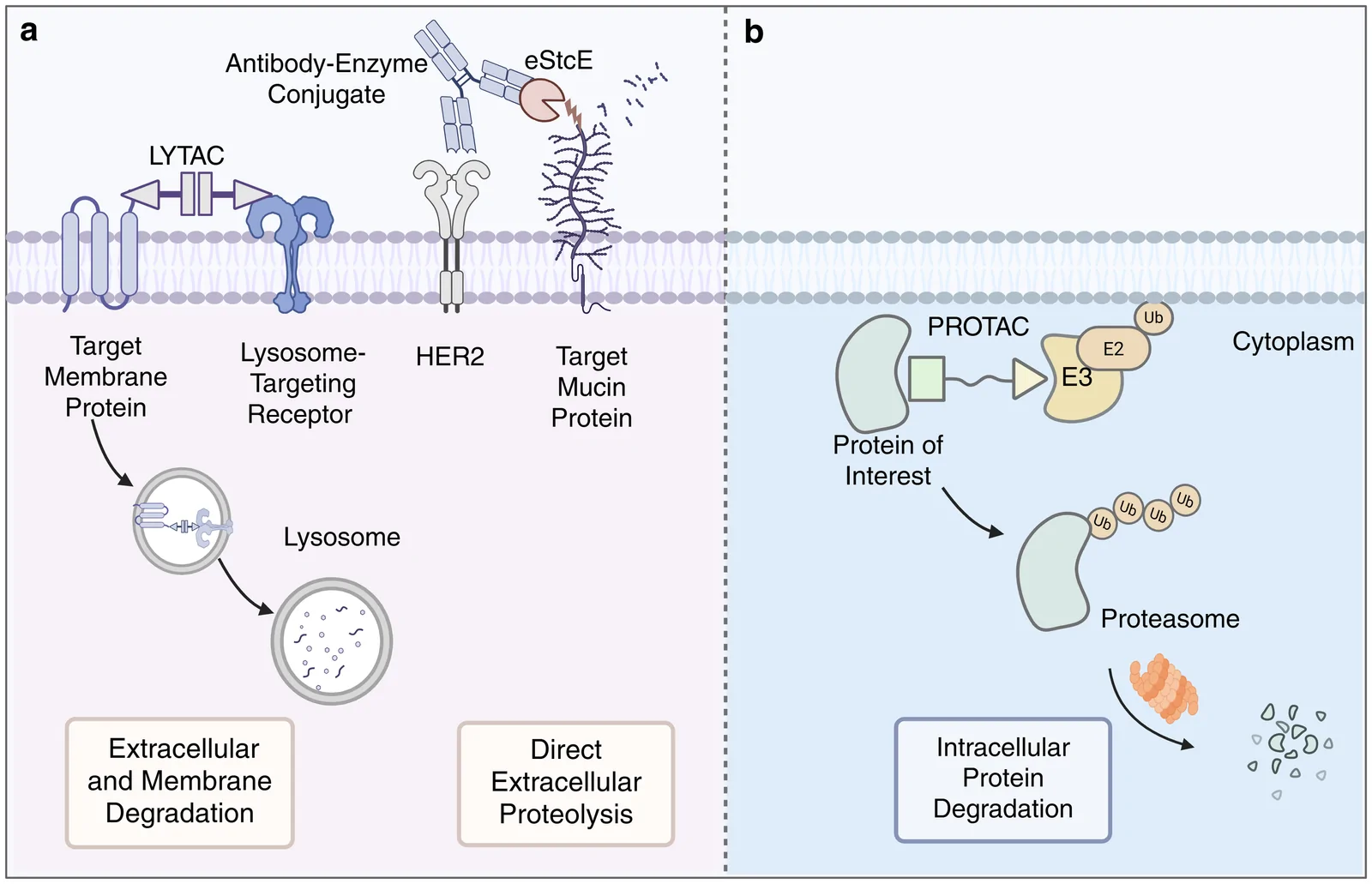
TPD modality overview. **(a)** LYTACs mediate lysosomal degradation of membrane proteins; antibody–enzyme conjugates enable direct extracellular proteolysis (e.g., eStcE targeting HER2 and mucins). **(b)** PROTACs recruit E3 ligases (with E2 enzymes) to ubiquitinate intracellular POIs for proteasomal degradation. Ub, ubiquitin.

**Figure 2:**
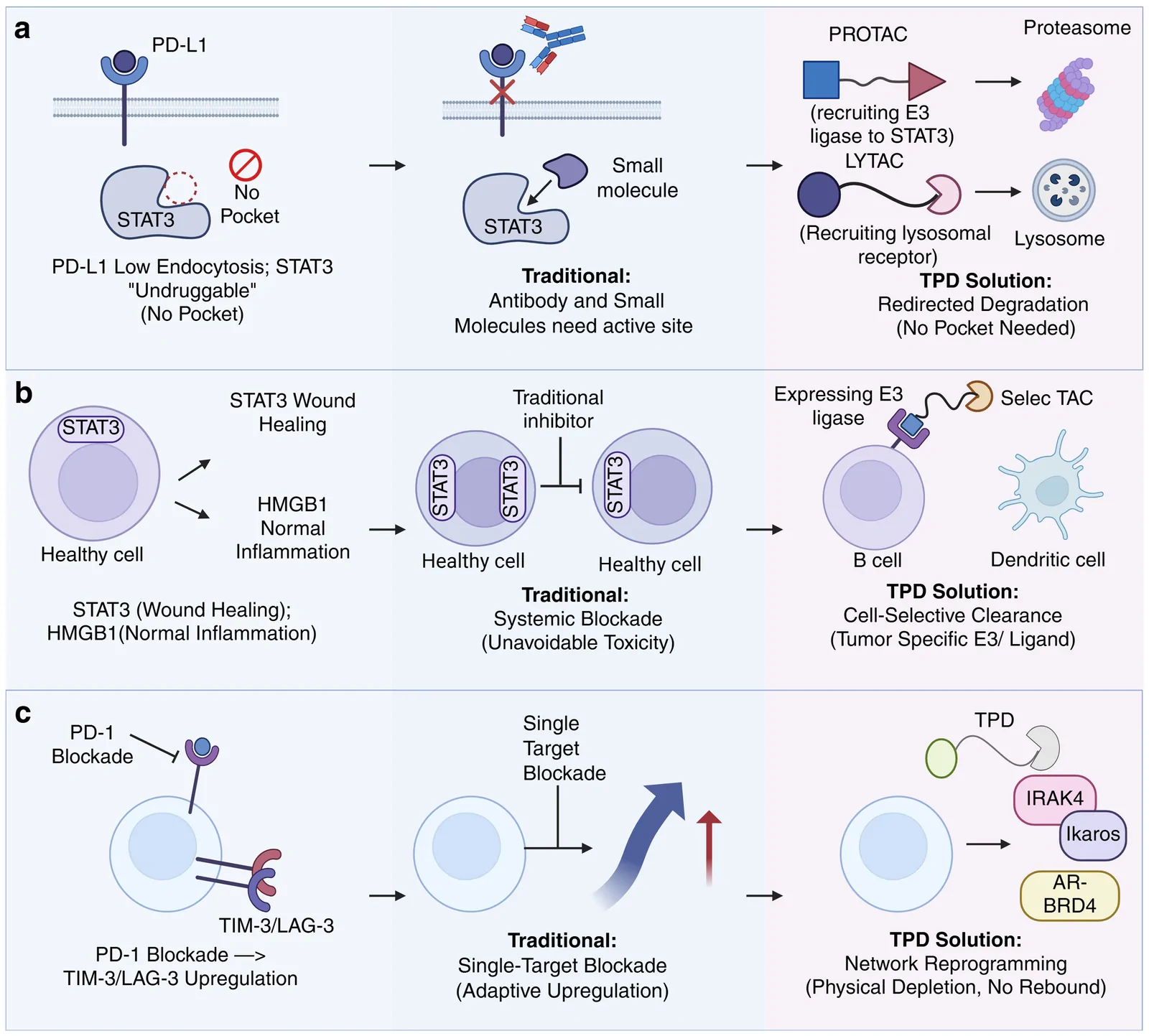
Mechanistic advantages of TPD over conventional inhibition. **(a)** Spatial and structural limits: conventional antibodies and small molecules depend on target endocytosis or druggable pockets, failing against PD-L1 (low endocytosis) and ‘undruggable’ STAT3 (no pocket); TPD bypasses these constraints via bifunctional degraders — PROTACs recruit E3 ligases for proteasomal elimination, and LYTACs redirect membrane proteins to lysosomes — enabling degradation irrespective of the target’s intrinsic properties (e.g., KT-333 degrades STAT3; LYTACs overcome PD-L1 endocytosis barriers). **(b)** Pleiotropic toxicity: systemic inhibition of pleiotropic targets (e.g., STAT3, HMGB1) disrupts physiological functions in healthy tissue; TPD achieves cell-selective clearance via tumor-restricted E3 ligases or homing ligands (e.g., SelecTACs), sparing normal tissue. **(c)** Adaptive resistance: single-target blockade (e.g., anti–PD-1) triggers compensatory upregulation of TIM-3/LAG-3; TPD prevents rebound signaling through physical protein depletion rather than transient inhibition (e.g., KT-413 co-degrades IRAK4 and Ikaros; RIPTAC HLD-0915 functionally sequesters AR–BRD4 to disrupt resistance networks).

The core of TPD technology directly addresses the spatial, structural, and functional barriers that render immunotherapeutic targets “undruggable.” By generating bifunctional molecules that simultaneously engage a target protein and an endogenous degradation effector, for instance, UPS or LTR,TPD catalyzes the selective elimination of disease-causing proteins rather than their reversible inhibition (11) (**Figure 1**). This mechanism overcomes spatial constraints by enabling degradation of membrane proteins regardless of endocytic efficiency, neutralizing secreted factors at their tumor source, and accessing intracellular hubs without requiring membrane permeability of the effector itself. Critically, TPD’s event-driven catalytic cycle—one degrader molecule clears multiple target copies—achieves profound protein depletion even at low concentrations, bypassing the occupancy limits of antibodies and the poor bioavailability of small molecules. For functionally pleiotropic targets, TPD offers a mechanistically distinct advantage: when coupled with tumor-homing ligands, degraders achieve cell-type-restricted clearance, sparing immune homeostasis in normal tissues while silencing tumor-intrinsic signaling. Moreover, by physically removing entire protein pools, TPD prevents adaptive resistance (11, 12), while inhibition of programmed cell death protein 1 (PD-1) and programmed death-ligand 1 (PD-L1) may trigger compensatory upregulation of T cell immunoglobulin and mucin domain-containing protein 3 (TIM-3) and lymphocyte activation gene 3 (LAG-3), TPD-mediated degradation of PD-L1 can abolish this signal persistently, reducing the drive for compensatory escape. Furthermore, TPD agents can be engineered to concurrently degrade both primary and compensatory checkpoints. This reprogramming of endogenous degradation machineries thus expands the druggable target space from a limited set of “inhibitable” nodes to the full spectrum of immunomodulatory proteins, irrespective of their subcellular location or structural “undruggability” (3) (**Figure 2**).

Strictly speaking, the term “undruggable” was coined to describe proteins that could not be targeted pharmacologically; however, progress is being made to “drug” many such targets, and more appropriate terms might therefore be “difficult to drug” or “yet to be drugged” (13). Clinically approved immune-checkpoint inhibitors targeting PD-1/PD-L1 have demonstrated dramatic efficacy in cancer patients (14), and the CD47-SIRPα axis has emerged as a promising target in cancer immunotherapy (15). For these membrane proteins, therefore, the rationale for TPD is not that antibodies cannot bind them, but that degradation achieves a mechanistically distinct outcome: unlike classic occupancy-based drugs, targeted protein degradation eliminates the target and works catalytically (16), and extracellular TPD strategies degrade membrane proteins by trafficking them to the lysosome (16), as exemplified by LYTACs and AbTACs that deliver cell-surface proteins such as PD-L1 for lysosomal degradation (7, 8). Antibody blockade is reversible and leaves the target available for re-engagement after antibody dissociation or clearance, whereas degradation physically removes the entire protein—a distinction central to the classification of these membrane proteins (16). For membrane receptors that continuously recycle between the cell surface and endosomes, lysosomal degradation can durably remove the signaling-competent pool, whereas antibody blockade leaves the receptor to reappear once the antibody dissociates or is cleared (7, 16). The distinct biological consequences of complete physical removal of the target—rather than transient blockade—thus constitute the rationale for applying TPD to membrane immune checkpoints.

### 2.2 TPD for degrading extracellular and membrane proteins

For proteins localized to the plasma membrane or secreted into the extracellular milieu, two distinct technological platforms have evolved, differentiated by their engagement of cellular degradation machinery versus direct enzymatic hydrolysis (**Figure 1**).

Degrading extracellular and membrane proteins that are difficult to target with conventional drugs has emerged as an alternative strategy by leveraging the endosome-lysosome pathway. Representative technologies in this field include LYTAC, Glycan-linked Targeting Chimera (GlueTAC), AbTAC, and Kinetokine Targeting Chimera (KineTAC) (12). Taking LYTAC as an example, its design ingenuity lies in constructing a bifunctional molecule that simultaneously binds to the extracellular domain of a target membrane/extracellular protein and a cell-surface LTR. The formation of this ternary complex triggers clathrin-mediated endocytosis, internalizing the entire complex and ultimately delivering the target protein to the lysosome for complete degradation (17). This provides a powerful new tool for degrading targets such as PD--L1 that are difficult to inhibit with small molecules.

Beyond the core LYTAC, GlueTAC, AbTAC and KineTAC platforms, the repertoire for degrading extracellular and membrane proteins has expanded substantially. Among lysosome-trafficking chimeras, soluble LYTACs (sLYTACs) (18), transferrin receptor-targeting chimeras (TransTACs) (19), integrin-facilitated lysosomal degradation (IFLD) (20), folate receptor-targeting chimeras (FRTACs) (21), ASGPR-driven nano-LYTACs (22), dual-receptor LYTACs (23), aptamer-based LYTACs (AptLYTACs) (24) and cell-penetrating peptide-based TACs (CPPTACs) (25) extend lysosomal degradation to additional membrane and extracellular targets. In parallel, antibody-based strategies—proteolysis-targeting antibodies (PROTABs) that tether the cell-surface E3 ubiquitin ligase zinc- and ring finger 3 (ZNRF3) to transmembrane proteins, resulting in target degradation both *in vitro* and *in vivo (*26), and antibody–enzyme conjugates that provide a direct extracellular hydrolysis strategy for heavily glycosylated targets such as mucins (27)—broaden the degradable surface proteome. Each platform carries distinct limitations—for example, AptLYTACs are constrained by nuclease degradation and rapid renal clearance (28), and TransTACs rely on the broadly expressed transferrin receptor for uptake (19). Overall, the degradable proteome now extends beyond intracellular proteins to demanding locations such as the extracellular matrix and cellular membranes (12). Within the UPS-dependent category, monovalent degraders—selective estrogen receptor degraders (SERDs), molecular glues (MGs), PROTACs, specific and nongenetic IAP-dependent protein erasers (SNIPERs) and hydroxy-tag-mediated degradation (HyT)—remain the core technologies (29, 30). Outside the UPS, intracellular degradation is mediated by autophagy-based degraders—autophagy-targeting chimeras (AUTACs) (31), autophagy-tethering compounds (ATTECs) (32) and AUTOphagy-TArgeting Chimeras (AUTOTACs) (33)—as well as by antibody-based Trim-Away, which enables acute and rapid depletion of endogenous intracellular proteins (34), and by in-cell click-assembled degraders (CLIPTACs), which allow intracellular formation of active PROTACs from cell-permeable precursors (35). Complementary to these degradation-centered approaches, deubiquitinase-targeting chimeras (DUBTACs) stabilize target proteins by shielding them from ubiquitin-dependent turnover (36).

Unlike mechanisms that depend on intracellular endocytosis, antibody-enzyme conjugates offer a distinct approach by providing a direct hydrolysis strategy (27). For instance, Pedram et al. developed a technology that involves genetically fusing a bacterially derived mucinase (eStcE) with a nanobody targeting Human Epidermal Growth Factor Receptor 2 (HER2). When the conjugate is densely enriched on the surface ofHER2-positive target cells via its antibody moiety, the local concentration of the enzyme is dramatically increased, enabling efficient and selective on-site cleavage and removal of mucins on those cells. In contrast, for “bystander” cells that do not express HER2, the enzyme fails to accumulate effectively, leaving their proteins intact (27). This extracellular proteolysis mode follows the principle of “effective molarity” and provides a complementary and powerful tool for degrading proteins with low endocytic efficiency or whose functions rely primarily on large extracellular domains, such as highly glycosylated mucin family members (27) (**Figure 1**). Collectively, these advances represent a substantial technical advance in overcoming the long-standing challenges of structural localization and dynamic elimination for extracellular targets, thereby transforming the vast extracellular immunomodulatory proteome into a repertoire of actionable therapeutic targets.

### 2.3 TPD for degrading intracellular proteins

The proliferation, survival, and immune evasion capabilities of tumor cells are highly dependent on the functions of numerous intracellular proteins. To target these proteins, UPS-based degradation strategies have shown remarkable potential. The primary technologies in this category include monovalent degraders, such as selective estrogen receptor degrader (SERD), molecular glues (MG), PROTAC, Specific and Nongenetic IAP-dependent Protein ERasers (SNIPER), and Hydroxy-Tag-mediated degradation (HyT) (12). Among these, PROTAC technology is particularly well-developed. PROTACs are bifunctional molecules that function as molecular matchmakers: one arm engages a disease-causing protein, while the other recruits an endogenous protein destruction complex. By forcing these two partners into proximity, PROTACs trigger a catalytic destruction signal that eliminates the target (37). This technology has paved the way for eliminating traditionally “undruggable” key oncogenic drivers (**Figure 1**), such as the transcription factor STAT3.

Molecular glues and PROTACs represent two complementary branches of targeted protein degradation that differ mechanistically: PROTACs are heterobifunctional small molecules that simultaneously engage a target protein and an E3 ligase to induce selective degradation through a catalytic mechanism, whereas molecular glues are low-molecular-weight small molecules that act as a molecular bridge, facilitating interaction between a target protein and an E3 ubiquitin ligase to enable degradation without requiring classical binding pockets (38). The concept of ‘molecular glues’ that induce neo-protein–protein associations was first revealed by cyclosporin A and FK506 (39), and molecular glue degraders operate through monovalent architectures that induce protein–protein interactions between E3 ligases and neosubstrates, including targets traditionally considered undruggable (40). Two aspects of this comparison deserve emphasis. At the mechanism level, molecular glues and PROTACs both induce E3-ligase-mediated degradation but differ in architecture and properties: PROTACs are heterobifunctional, whereas molecular glues are monovalent and typically smaller, offering advantages in chemical simplicity and cell permeability, although both modalities must contend with potential CRBN neosubstrate liabilities (38, 40, 41). The broader cellular context in which degradation operates—including dynamic regulatory layers and structural biology considerations—is discussed in the Outlook section.

The chemical stability of the linker is a major determinant of the systemic behavior of bifunctional degraders: the chemical nature and length of the linker play a major role in the metabolic liability of degraders, and their metabolism cannot simply be predicted from that of their constituent ligands (42). An effective linker should maintain sufficient stability in the circulation to minimize premature cleavage or degradation before the degrader reaches its intended site of action. Susceptibility to serum proteases, esterases and other metabolic enzymes can reduce the exposure of intact degrader molecules and thereby compromise pharmacological activity. Accordingly, serum and plasma stability should be incorporated into linker optimization together with metabolic stability, permeability and degradation potency. Experience from antibody–drug conjugate development provides useful principles for evaluating linker stability, although the relevant stability requirements differ among degrader modalities (43, 44). Building on this principle, TPD has adopted two complementary approaches based on modular “click chemistry” (35, 45). The first is synthetic: bioorthogonal click ligation—for example, copper-catalyzed azide–alkyne cycloaddition—enables the rapid synthesis of degraders from azide- and alkyne-functionalized precursors, providing an efficient platform for degrader discovery and structure–activity optimization (45); this approach improves synthetic modularity rather than serum stability. The second is functional: in-cell click self-assembly (e.g., CLIPTAC), in which the two halves of a degrader are delivered separately and ligated intracellularly, allows the active bifunctional species to be formed inside cells from smaller precursors (35). However, click chemistry itself does not inherently improve serum stability, and the serum and metabolic stability of both the precursor molecules and the assembled degrader must still be evaluated independently.

The canonical bifunctional degrader is a heterobifunctional small molecule that simultaneously engages a target protein and an E3 ligase to induce selective degradation through a catalytic mechanism (38). Induced polymerization represents a mechanistically distinct concept, in which a small molecule induces the highly specific, reversible polymerization of a target protein, followed by its sequestration into cellular foci and subsequent degradation (46). The paradigm example is BI-3802, which binds to the BTB domain of the oncogenic transcription factor BCL6 and leads to its proteasomal degradation (46); drug-induced formation of BCL6 filaments facilitates ubiquitination by the SIAH1 E3 ubiquitin ligase (46). Induced polymerization therefore does not bypass the ubiquitin-proteasome system: by promoting target self-assembly, it can increase the efficiency of E3-mediated ubiquitination, but it does not remove the requirement for an E3 ligase. Accordingly, induced polymerization should not be regarded as a general alternative for proteins that cannot be ubiquitinated efficiently by existing E3 ligases; rather, it exemplifies how modulating the oligomeric state of a target can improve its susceptibility to ubiquitination and degradation.

## 3 Reversing primary immune evasion

The immunological consequences of TPD should not be treated as a uniform class effect, as the evidence supporting immune reprogramming differs substantially across targets. The examples that follow illustrate three evidentiary tiers: (i) direct degradation — target protein elimination validated biochemically in cells or tumors; (ii) downstream immune modulation — functional immune changes (e.g., T-cell activation, macrophage phagocytosis) demonstrated in preclinical models; and (iii) clinically proven immunotherapy sensitization — improved response to checkpoint blockade or endocrine therapy in patients. Throughout the text, clinical trial registration numbers (NCT identifiers) serve as the primary indicator of tier iii evidence.

**Figure 3:**
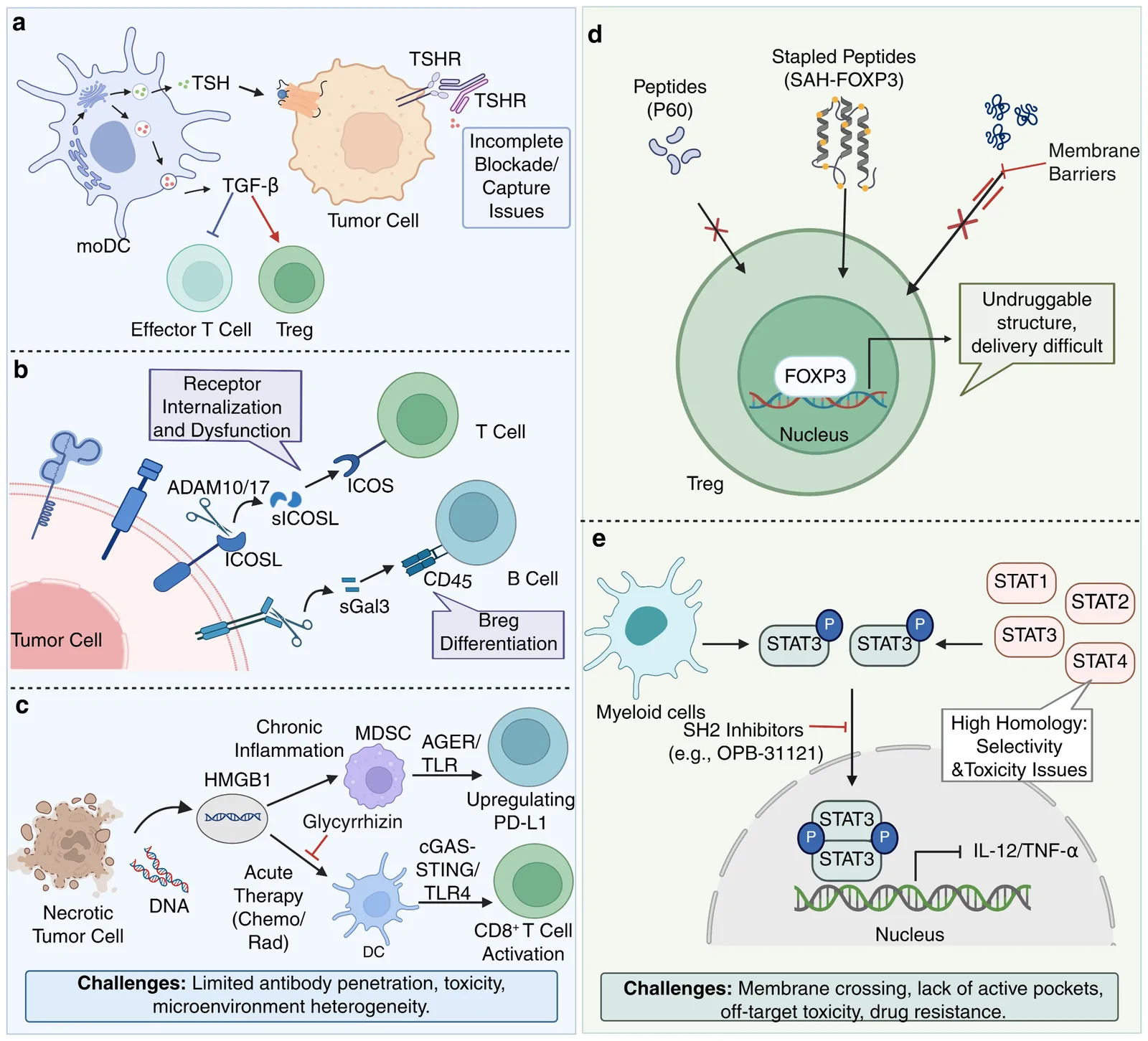
Extracellular and intracellular immunotherapy targets and associated challenges. **(a)** Direct secretion: moDC-derived TSH and TGF-β modulate tumor cells (via TSHR) and effector/regulatory T cells; the anti–PD-L1/TGF-β trap M7824 incompletely captures TGF-β ligands. **(b)** Membrane shedding: ADAM10/17 cleaves ICOSL to sICOSL (inducing T cell receptor internalization and dysfunction) and generates sGal3 (binding CD45 to drive Breg differentiation). **(c)** Stress-induced outflow: necrotic tumor cells release HMGB1; chronic inflammation activates MDSCs (upregulating PD-L1 via AGER/TLR), whereas acute therapy (chemotherapy or radiation) activates DCs via cGAS–STING/TLR4 to promote CD8^+^ T cell activation. Glycyrrhizin inhibits HMGB1 signaling. **(d)** Nuclear targets: FOXP3-binding peptides (P60, SAH-FOXP3) face membrane barriers, undruggable structure and delivery difficulty. **(e)** Cytoplasmic signaling: p-STAT3 is targeted by SH2-domain inhibitors (e.g., OPB-31121), but homology among STAT1/2/3/4 causes selectivity and toxicity issues; persistent p-STAT3 suppresses IL-12 and TNF-α. Key challenges include limited antibody penetration, off-target toxicity and microenvironment heterogeneity for extracellular targets, and membrane crossing, lack of druggable pockets, resistance and off-target effects for intracellular targets.

Based on their molecular localization, immunotherapy targets can be divided into three major categories: intracellular targets — for example, forkhead box P3 (FOXP3) and STAT3; membrane-localized targets — for example, PD-L1; and extracellular targets. Extracellular targets can be further classified into three subtypes according to their origin: proteins directly secreted into the extracellular space, such as transforming growth factor-beta (TGF-β) and thyroid stimulating hormone (TSH); membrane proteins shed after proteolytic cleavage, such as soluble inducible T-cell co-stimulator ligand (sICOSL), soluble galectin-3 (sGal3) and soluble glucose-regulated protein 78 (sGRP78); and cytoplasmic proteins released under stress conditions, such as high mobility group box 1 (HMGB1) and extracellular heat shock protein 70 (eHSP70) (47, 48) (**Figure 3**).

### 3.1 Dismantling membrane adaptive regulators

To address challenges such as adaptive upregulation and heterogeneity of PD--L1 in cancer immunotherapy, multiple novel degradation strategies have emerged. For example, using the KineTAC platform, a bispecific antibody named CXCL12–Atz targeting PD--L1 was developed. Through its CXCL12 arm, it recruits the endogenous decoy recycling receptor C-X-C motif chemokine receptor 7 (CXCR7), directing PD--L1 into the lysosomal degradation pathway. This approach achieved approximately 70% degradation efficiency of PD--L1 in MDA--MB-231 cells, accompanied by restored T-cell cytotoxicity and reduced tumor growth in xenograft models (49). Furthermore, several other innovative designs have demonstrated superior anti-tumor efficacy compared to traditional antibodies inpreclinical studies (12): the chimeric peptidePD--LYSO, which fuses a PD--L1--binding domain with a lysosomal sorting signal of huntingtin-interacting protein 1-related (HIP1R), directly escorts the target protein to the lysosome (50). The PROTAC molecule P22 combines PD-1/PD-L1 inhibitory activity with lysosome--dependent PD--L1 degradation, and its capacity to restore T-cell immune responses surpasses that of Keytruda (51). Leveraging the high expression of integrins in tumors, the Integrin--Facilitated Lysosomal Degradation (IFLD) strategy employs bifunctional degraders that link a target protein ligand with an integrin ligand. Its representative degrader, BMS--L1-RGD, achieves highly efficient degradation of PD--L1 (20). AC-1, a representative AbTAC, recruits the membrane--associated E3 ligase ring finger protein 43 (RNF43), effectively inducing lysosomal degradation of PD--L1 (8).

**Figure 4:**
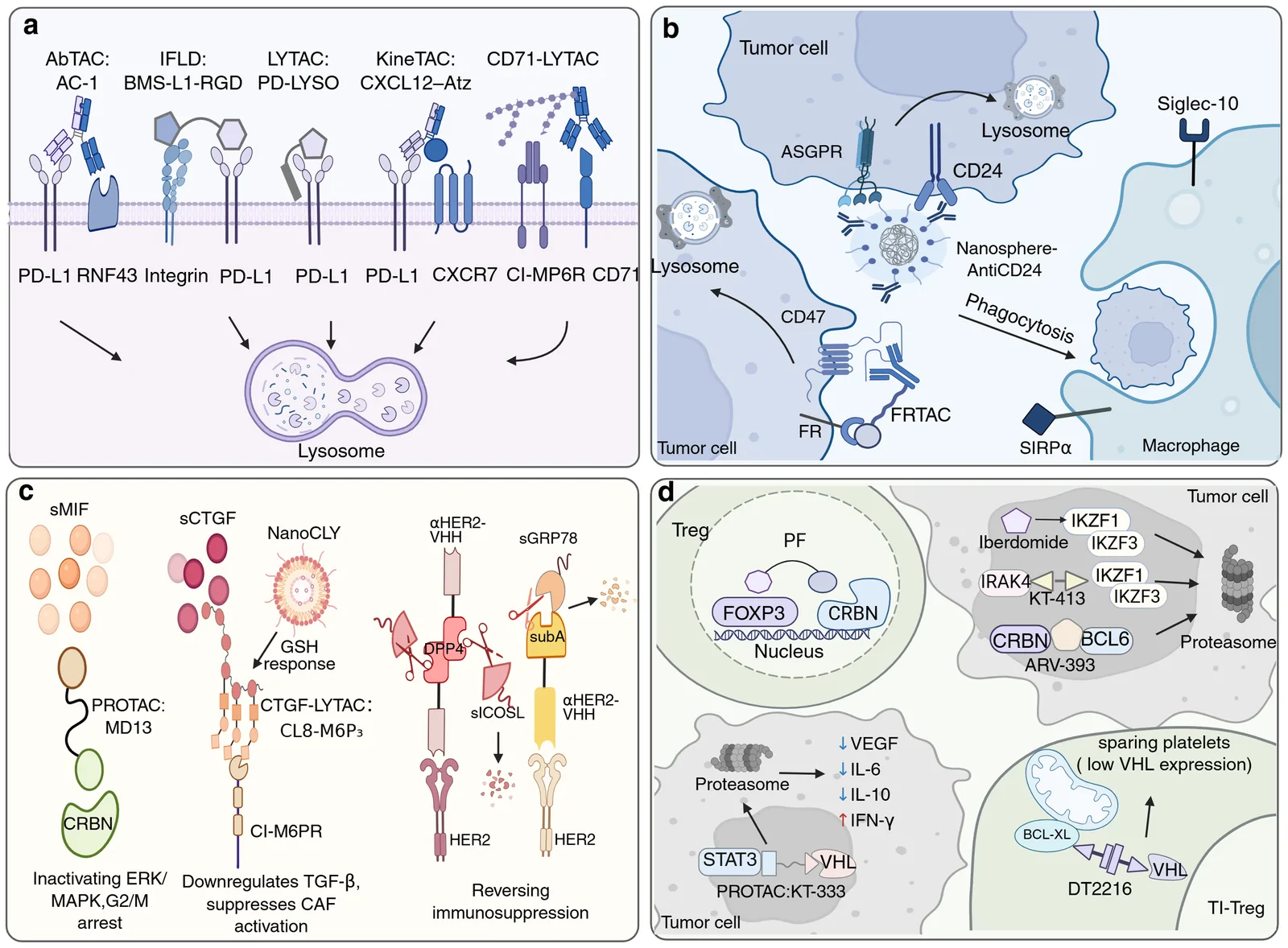
TPD reverses primary immune evasion by dismantling membrane, soluble and intracellular immunosuppressive nodes. **(a)** Membrane adaptive regulators: lysosome-directed chimeras — including AbTACs (AC-1 recruiting RNF43), IFLD (BMS-L1-RGD engaging integrins), LYTACs (PD-LYSO via HIP1R sorting), KineTACs (CXCL12–Atz recruiting CXCR7) and CD71-directed LYTACs (engaging CI-M6PR) — degrade PD-L1 and CD71 to restore T cell cytotoxicity. **(b)** Membrane innate checkpoints: ASGPR-driven LYTACs (Nanosphere-AntiCD24) and FR-targeting chimeras (FRTACs) degrade CD24 and CD47, respectively, restoring macrophage phagocytosis. **(c)** Soluble immunosuppressive signals: the CRBN-recruiting PROTAC MD13 degrades MIF, inactivating ERK/MAPK signaling; the LYTAC CL8-M6P3 (delivered via the glutathione-responsive nanoplatform NanoCLY) degrades CTGF to suppress CAF activation; and nanobody–enzyme fusions (anti-HER2-VHH conjugated to DPP4 or SubA) degrade sICOSL and sGRP78 to reverse immunosuppression. **(d)** Intracellular transcriptional brakes and survival factors: the CRBN-recruiting PROTAC PF degrades FOXP3 in Tregs; the VHL-recruiting PROTAC KT-333 degrades STAT3, downregulating VEGF, IL-6 and IL-10 while upregulating an IFN-γ-related gene signature; CRBN modulators (iberdomide) target IKZF1/IKZF3, and the bifunctional degrader KT-413 co-degrades IRAK4 and IKZF1/3; the VHL-recruiting PROTAC DT2216 selectively eliminates BCL-XL in TI-Tregs while sparing platelets owing to their low VHL expression.

In a similar vein, the degradation machinery has been redirected toward membrane proteins governing cellular metabolism and iron uptake, a critical nexus for proliferating cancer cells. For example, the transferrin receptor, cluster of differentiation 71 (CD71), has been redirected to lysosomal degradation by LYTACs. Upregulation of CD71 marks T-cell activation and correlates with their proliferative activity (52). LYTAC targeting CD71 utilizes a bispecific architecture that conjugates a CD71--binding antibody with a glycan ligand that engages the cation-independent mannose 6-phosphate receptor (CI-M6PR) (7). This design successfully redirects CD71—which normally recycles between the cell surface and early endosomes—into the lysosomal degradation pathway, achieving over 80% degradation of CD71 in Jurkat T-cell leukemia cells. This degradation effectively blocks cancer cells’ ability to take up transferrin via CD71, demonstrating the potential of the LYTAC platform for clearing traditionally challenging membrane proteins (7) (**Figure 4**).

### 3.2 Dismantling membrane innate checkpoints

Having explored adaptive immune checkpoints, we now turn to the innate arm of the immune system, where membrane-bound “don’t eat me” signals present formidable barriers to effective tumor clearance. Cluster of differentiation 24 (CD24) is a membrane protein overexpressed in various solid tumors, including hepatocellular carcinoma (HCC), that mediates immune evasion by delivering a “don’t eat me” signal via interaction with macrophage sialic acid-binding immunoglobulin-type lectin 10 (Siglec-10) (53). Current therapies achieve only transient signaling blockade without eliminating CD24. To overcome this, researchers developedNanosphere-AntiCD24, anasialoglycoprotein receptor (ASGPR) -driven LYTAC platform that conjugates anti-CD24 antibodies to N-acetylgalactosamine (GalNAc) self-assembled nanospheres, leveraging liver-specific ASGPR to mediate internalization and lysosomal degradation of the target protein. Degradation was inhibited by ASGPR knockout or exogenous GalNAc, confirming ASGPR dependency; functionally, CD24 degradation enhanced macrophage phagocytosis by blocking the CD24/Siglec-10 pathway. *In vivo*, Nanosphere-AntiCD24 showed better stability than the free antibody with no observable liver toxicity (22).

A parallel innate checkpoint, the cluster of differentiation 47 (CD47)/signal regulatory protein alpha (SIRPα) axis, has similarly drawn intense interest, though its therapeutic targeting has been plagued by systemic toxicities that TPD strategies are uniquely positioned tocircumvent. CD47 is a transmembrane proteinwidely expressed on the surface of tumor cells. By engaging the SIRPα receptor on myeloid cells (including macrophages, dendritic cells, and neutrophils), it delivers a “don’t eat me” signal that inhibits phagocytosis. The CD47/SIRPα axis not only acts as an innate immune checkpoint but also indirectly influences adaptive immune responses by modulating the function of myeloid cells (54). However, current monoclonal antibodies targeting the CD47–SIRPα axis and antigen sink effects. Researchers developed folate receptor-targeting chimeras (FRTACs), a platform that employs folic acid (FA) as a ligand to construct bifunctional molecules through conjugation with an anti-CD47 antibody. The resulting CD47–antibody complex undergoes cellular internalization via folate receptor (FR)-mediated endocytosis, taking advantage of the high FR expression on tumor cell surfaces, and is subsequently routed to the lysosomal pathway for degradation. Treatment with 100 nM FRTAC for 24 hours resulted in significant degradation of CD47 protein in MDA-MB-231 and A549 cells (21) (**Figure 4**).

### 3.3 Clearing soluble immunosuppressive signals

Beyond the constraints of the cell membrane, the tumor microenvironment is awash in soluble factors that propagate immunosuppression and resistance, demanding a different set of degradation tools. Macrophage migration inhibitory factor (MIF) regulates the immunosuppressive effects of glucocorticoids and governs cell survival pathways. As a crucial upstream mediator of both innate and adaptive immunity as well as host survival pathways, MIF plays protective roles in pathogen clearance, yet it can also drive detrimental inflammation and promote cancer progression (55). MIF directly modulates tumor immune responses by inducing T helper 2 (Th2) and T helper 17 (Th17) expansion, suppressing cytotoxic T lymphocyte (CTL) activation, and promoting the tumor infiltration and immunosuppressive activity of myeloid-derived suppressor cells (MDSCs). Its expression correlates positively with cancer phenotype severity, and its function depends on interaction with the cell-surface receptors cluster of differentiation 74 (CD74) and cluster of differentiation 44 (CD44) (56). The MIF-targeting PROTAC degrader MD13, leveraging the cereblon (CRBN) E3 ligase and the proteasome pathway, achieves efficient MIF degradation in non-small cell lung cancer (NSCLC) A549 cells (DC_50_ ≈ 100 nM). Clearance of MIF directly inactivates its downstream extracellular signal-regulated kinase (ERK)/mitogen-activated protein kinase (MAPK) signaling pathway and triggers G_2_/M phase cell cycle arrest. This mechanism ultimately translates into a marked anti-proliferative effect in *in vitro* models (57).

Similarly, the complex crosstalk between tumor cells and the surrounding stroma generates soluble mediators such as connective tissue growth factor (CTGF), which has proven recalcitrant to conventional antibody blockade. A CTGF/IL-6 positive feedback loop exists between triple-negative breast cancer (TNBC) cells and cancer-associated fibroblasts (CAFs): TNBC cells secrete CTGF to activate inflammatory CAFs, which in turn secrete IL-6 to further upregulate CTGF expression. Conventional anti-CTGF antibody (FG-3019) targets only a single domain, and CTGF is susceptible to protease cleavage, with the released fragments retaining pro-tumor activity, resulting in limited efficacy. To address this, researchers developed a peptide-based LYTAC (CL8-M6P3) that engages CI-M6PR to mediate CTGF internalization and lysosomal degradation, and further constructed a glutathione-responsive self-assembling nanoplatform, NanoCLY. Complete degradation of CTGF downregulates TGF-β signaling, disrupts CTGF-IL-6 crosstalk, and suppresses inflammatory CAF activation. In TNBC-bearing mice, NanoCLY monotherapy achieved a tumor growth inhibition rate of 59.62%, with enhanced efficacy when combined with paclitaxel, and effectively suppressed lung and bone metastasis, demonstrating significantly superior anti-tumor activity compared to FG-3019 (58).

Following ectodomain shedding by a disintegrin and metalloproteinase (ADAM) family proteases, membrane proteins generate soluble immune checkpoint molecules. Recent studies have identified sICOSL, sGal3, and sGRP78 as key molecules. sICOSL induces inducible T-cell co-stimulator (ICOS) receptor internalization and degradation on T cells, leading to T-cell exhaustion. sGal3 binds cluster of differentiation 45 (CD45) to activate STAT3, promoting immunosuppressive B cell differentiation and IL-10 production. sGRP78 modulates toll-like receptor 4 (TLR4) signaling to induce myeloid cell death and drives B cells toward an immunosuppressive phenotype (59–62). Serum levels of these soluble receptors correlate with disease severity (63). However, existing strategies have limitations: EZH2 and ADAM10/17 inhibitors targeting sICOSL lack tissue specificity; the sGal3 inhibitor TD139 exhibits nonspecific binding to galectin 1; and no effective therapy is available for sGRP78. To address these issues, a modular nanobody-enzyme fusion platform has been developed for targeted protein degradation of these factors. The platform fuses anti-HER2 or anti-trophoblast cell surface antigen 2 nanobodies with effector enzymes: dipeptidyl peptidase 4 (DPP4) degrades sICOSL, and subtilase cytotoxin subunit A (SubA) protease degrades sGRP78. Through targeted anchoring and local degradation, this platform specifically clears soluble factors in the tumor microenvironment, reverses immunosuppression, and enhances chemotherapy efficacy in animal models. By exchanging targeting and enzymatic modules, this modular platform can be adapted to degrade various soluble immune checkpoints, offering a versatile solution for reversing tumor immunosuppression.

Degrader-antibody conjugates (DACs)—novel entities that combine a PROTAC payload with a monoclonal antibody via a chemical linker (64)—represent an antibody-based strategy directly relevant to immune-microenvironment modulation. Proof of concept for the antibody–payload architecture has been provided mainly for intracellular targets: antibody–PROTAC conjugates achieve HER2-dependent degradation of BRD4 in cancer cells (65), and an antibody–PROTAC conjugate co-suppresses the BRD4/c-Myc axis and downstream PD-L1 expression in triple-negative breast cancer cells (66). Antibody-mediated delivery has also been extended to chimeric degraders targeting estrogen receptor alpha (ERα): degrader-antibody conjugates comprising distinct ERα-targeting degrader entities and multiple ADC-type linker modalities achieve antigen-dependent delivery of the degrader payload (67). For immunosuppressive factors, the most directly relevant antibody-based degraders act on the cell surface. AbTACs—genetically encoded bispecific antibodies that recruit the membrane-bound E3 ligase RNF43—induce lysosomal degradation of cell-surface proteins such as PD-L1 (8, 68), and rational protein engineering has optimized this platform for efficient degradation of PD-L1 and EGFR (69). Likewise, proteolysis-targeting antibodies (PROTABs) that tether the cell-surface E3 ligase ZNRF3 to transmembrane proteins achieve target degradation *in vitro* and *in vivo (*26), and the tumor-specific expression of RNF43 and ZNRF3 in colorectal cancer confers lesion-selective degradation capability (26, 68). These examples illustrate how the precision of monoclonal antibodies can be combined with degradation-based pharmacology to eliminate membrane-associated immunosuppressive factors. Whether DACs can be extended to soluble extracellular immunosuppressive factors—such as immunosuppressive cytokines—with sufficient tumor selectivity remains to be demonstrated, and tumor-localized delivery, linker stability and release kinetics will require further optimization (**Figure 4**).

### 3.4 Eliminating intracellular transcriptional brakes and survival factors

Unlike membrane targets amenable to antibody blockade, intracellular transcription factors lack druggable pockets and are inaccessible to biologics. TPD offers a unique solution by repurposing the cell’s endogenous proteolytic machinery, bypassing the need for membrane permeability and active-site occupancy. Clearing extracellular and membrane-bound signals relieves immediate immunosuppression, but durable immune reprogramming demands dismantling the intracellular transcriptional machinery that sustains regulatory cell phenotypes. The intracellular transcription factor FOXP3 is a key regulator of regulatory T cell (Treg) function and is essential for Treg maintenance under pathological conditions (70). However, FOXP3 localizes to the nucleus, posing a formidable delivery barrier for conventional drugs (71). Various intervention strategies have been explored, each with fundamental limitations: early peptides required transfection for intracellular delivery (72); the P60 peptide binds FOXP3 but requires high concentration with significant cytotoxicity (73); epirubicin lacks pathway specificity and carries inherent cytotoxicity (74); the recently reported hydrocarbon-stapled peptide shows improved permeability and reduced toxicity, yet its overly rigid structure may compromise dynamic interaction mimicry, and its intracellular mechanism remains unclear (75). To address these challenges, Wang and colleagues developed a FOXP3-targeting PROTAC (PF). PF recruits CRBN via pomalidomide while binding FOXP3 through the P60 peptide, forming a ternary complex that marks FOXP3 for proteasomal degradation. The anti-tumor effect of PF is dependent onCD8^+^ T cell–mediated immunity. In the RENCA renal cancer mouse model, PF monotherapy significantly suppressed tumor growth; combination with anti-PD-1 antibody or an mTOR inhibitor further enhanced anti-tumor immunity. PF also reduced the proportion of FOXP3+ Tregs and enhanced CD8+ T cell function in human peripheral blood mononuclear cells (PBMCs) (76).

This intracellular targeting logic also applies to STAT3, a central signaling node in the tumor microenvironment that regulates dendritic cell (DC) function through interplay with signal transducer and activator of transcription 5 (STAT5) (77). Persistently activated STAT3impairs DC maturation by inhibiting IL-12 and TNF-α, suppressing innate immunity and mediating immune escape (78). STAT3 also upregulates vascular endothelial growth factor (VEGF), interleukin-6 (IL-6), and interleukin-10 (IL-10) via the IL-6/Janus kinase (JAK)/STAT3 axis, establishing an immunosuppressive microenvironment (79). Despite being a highly promising target, existing STAT3 inhibitors have notable limitations. The Src homology 2 (SH2) domain-targeting inhibitor OPB-31121 suffers from poor selectivity, off-target toxicity, and neurotoxicity due to high homology among STAT family SH2 domains, resulting in very low translation rates (80, 81). The peptidomimetic inhibitor S3I-M2001 has low binding affinity and inherent cytotoxicity (80, 82). The DNA-binding domain-targeting inhibitor CPA-7 shows poor blood-brain barrier permeability, while galiellalactone lacks sufficient specificity (80). These limitations highlight the need for novel STAT3 degradation strategies. KT-333 has demonstrated clear anti-tumor activity and immune--modulating effects in its Phase I trial (NCT05225584): three complete responses (CR) were observed in patients with classical Hodgkin lymphoma (cHL) and natural killer (NK) -cell lymphoma, along with four partial responses (PR) in patients with cutaneous T-cell lymphoma (CTCL) (6). Biomarker analyses confirmed >90% STAT3 degradation in both peripheral blood and tumor tissue, accompanied by upregulation of an interferon--γ (IFN-γ)--related gene signature (6).

Unlike classic occupancy-based drugs, they eliminate the target and act catalytically, which can enable pharmacodynamic effects beyond continuous target occupancy, although the duration of target suppression remains dependent on degrader exposure and target re-synthesis. A key pharmacological question is whether the catalytic turnover of degraders translates into superior sustained target suppression compared with high-affinity, long-acting monoclonal antibodies or small-molecule inhibitors (83). Degraders co-opt the ubiquitin-proteasome system to modulate protein concentrations at a post-translational level (16, 83). Accordingly, sustained loss of target without re-dosing is among the optimized parameters required of highly efficacious degraders (84). Consistent with these expectations, KT-333 produced mean maximal STAT3 degradation of up to 95% in peripheral blood mononuclear cells (with up to 97.5% maximal degradation) and induced an IFNγ-stimulated gene signature in tumor biopsies, demonstrating substantial target knockdown and pathway modulation in patients (6). The kinetic basis of this behavior is well established: the catalytic mechanism of PROTACs can, in principle, deliver pharmacodynamic efficacy that extends beyond the detectable pharmacokinetic presence of the compound, with the duration of this extended response driven by the synthesis rate of the target protein (85). The same kinetics also define the limits of sustained suppression: PROTAC withdrawal allows for the rapid restoration of target protein levels as fast as protein re-synthesis permits (83), and in rats, RIPK2 levels showed recovery and returned to baseline levels at the later 240 h time point after a single dose (85). Clinically, pharmacodynamic analysis of the IRAK4 degrader KT-474 showed that IRAK4 reduction reached its nadir by 48 h after a single dose and showed early signs of recovery 4 days later, with recovery continuing through day 14 (86). Thus, for continuously synthesized targets such as STAT3, the duration of suppression is set by the balance between the degradation rate and the resynthesis rate, and mechanism-based pharmacodynamic modeling that links mRNA and protein turnover has been applied to PROTAC pharmacodynamics (87). Because target levels re-accumulate as resynthesis permits after the degrader is cleared (83, 85, 86), the dosing interval must be aligned with target resynthesis kinetics, or complementary strategies employed, to maintain continuous suppression. In contrast to the continuous target occupancy maintained by long-acting antibodies, degrader-mediated suppression is intrinsically limited by target resynthesis once the agent is cleared (83, 85, 86). To date, no head-to-head clinical study has directly compared rebound kinetics between degraders and long-acting biologics, and the arguments presented here are therefore mechanistic and require prospective validation. Claims of a “catalytic advantage” should accordingly be made with pharmacokinetic/pharmacodynamic rigor rather than assumed.

Beyond these core oncogenic drivers, a suite of emerging degraders is now interrogating the transcription factors that govern lymphocyte fate and inflammatory signaling, with clinical translation already underway. Ikaros zinc finger 1 (IKZF1) and Aiolos (IKZF3) are key transcription factors governing lymphocyte development and function, and their abnormalities are closely associated with hematological malignancies (88). Iberdomide is a next-generation CRBN E3 ligase modulator that induces ubiquitination and degradation of Ikaros and Aiolos. Mechanistic studies show that iberdomide overcomes CRBN dysregulation associated with immunomodulatory imide drug (IMiD) resistance, stimulates immune cell proliferation, and induces a shift toward an activated/effector memory T cell phenotype (89). In a Phase I/II clinical trial (NCT02773030), iberdomide combined with dexamethasone achieved an overall response rate of 26%–32% in heavily pretreated multiple myeloma patients (median 5–6 prior lines) who were refractory to immunomodulatory agents (90). Meanwhile, interleukin-1 receptor-associated kinase 4 (IRAK4) serves as a central signaling node for pro-tumor inflammation in the tumor immune microenvironment, driving production of interleukin-1 beta (IL-1β) and IL-6 via nuclear factor kappa-light-chain-enhancer of activated B cells (NF-κB) activation downstream of interleukin-1 receptor (IL-1R) and Toll-like receptor (TLR) (91). KT-413, a bifunctional degrader targeting both IRAK4 and Ikaros/Aiolos, has completed preclinical studies and is currently being evaluated in a Phase I clinical trial (NCT05233033) for B-cell lymphoma (92).

B-cell lymphoma 6 (BCL6) offers a complementary node in this transcriptional network. BCL6 is a master transcriptional regulator of the germinal center B-cell response and also controls the stem--like versus terminally differentiated balance in CD8+ T cells, thereby impacting the durability of anti-tumor immunity and response to immunotherapy (93). ARV-393, a BCL6--targeting PROTAC degrader, is currently in Phase I clinical development for relapsed/refractory non--Hodgkin lymphoma (NCT06393738) (94).

This level of precision extends to the elimination of tumor-infiltrating Tregs, where TPD spares systemic counterparts in ways inhibitors cannot. Tumor-infiltrating regulatory T cells(TI-Tregs) are highly enriched in the tumor microenvironment, where they promote immune evasion by suppressing effector T cell function. BCL-2 extra-large (BCL-XL), a key survival factor for TI-Tregs, is specifically upregulated in these cells, making it a potential molecular target for selective TI-Treg elimination. However, conventional Treg-targeting strategies often induce autoimmune toxicity due to their inability to distinguish TI-Tregs from those in normal tissues, while direct inhibition of BCL-XL causes severe thrombocytopenia as an on-target, dose-limiting toxicity (95). To overcome these obstacles, researchers developed DT2216, a PROTAC degrader that selectively eliminates BCL-XL by recruiting the von Hippel-Lindau (VHL) E3 ligase, while sparing platelets owing to their low VHL expression. In preclinical models, DT2216 treatment effectively depleted TI-Tregs, activated tumor-infiltrating CD8^+^ T cells, and established an immunologically active tumor microenvironment without affecting Tregs in normal tissues (96). Currently, DT2216 has completed a Phase I clinical trial in patients with relapsed/refractory solid tumors (NCT04886622), and a Phase I/II study in combination with chemotherapy is ongoing (NCT06620302), positioning it as a potential strategy for patients who are resistant to existing immunotherapies (**Figure 4**).

## 4 Overcoming therapy resistance

TPD not only dismantles primary immune evasion but also overcomes therapy resistance. Based on the resistance mechanisms discussed below, we identify four scenarios where degradation offers a distinct advantage over inhibition: (i) target mutation abolishing inhibitor binding (e.g., EGFR C797S, ESR1 mutations) — degradation bypasses the mutated binding site; (ii) compensatory signaling reactivating the pathway (e.g., PI3K/AKT/mTOR) — physical removal prevents signal rebound; (iii) scaffolding or non-enzymatic functions (e.g., RIPK1, NAT10) — degradation eliminates all functions, not just catalytic activity; and (iv) secreted or shed factors with rapid turnover (e.g., eNAMPT, TGF-β) — degradation reduces the steady-state concentration of active species, whereas inhibition only neutralizes the ligand without reducing its production.

### 4.1 Degrading mutated and upregulated membrane proteins

**Figure 5:**
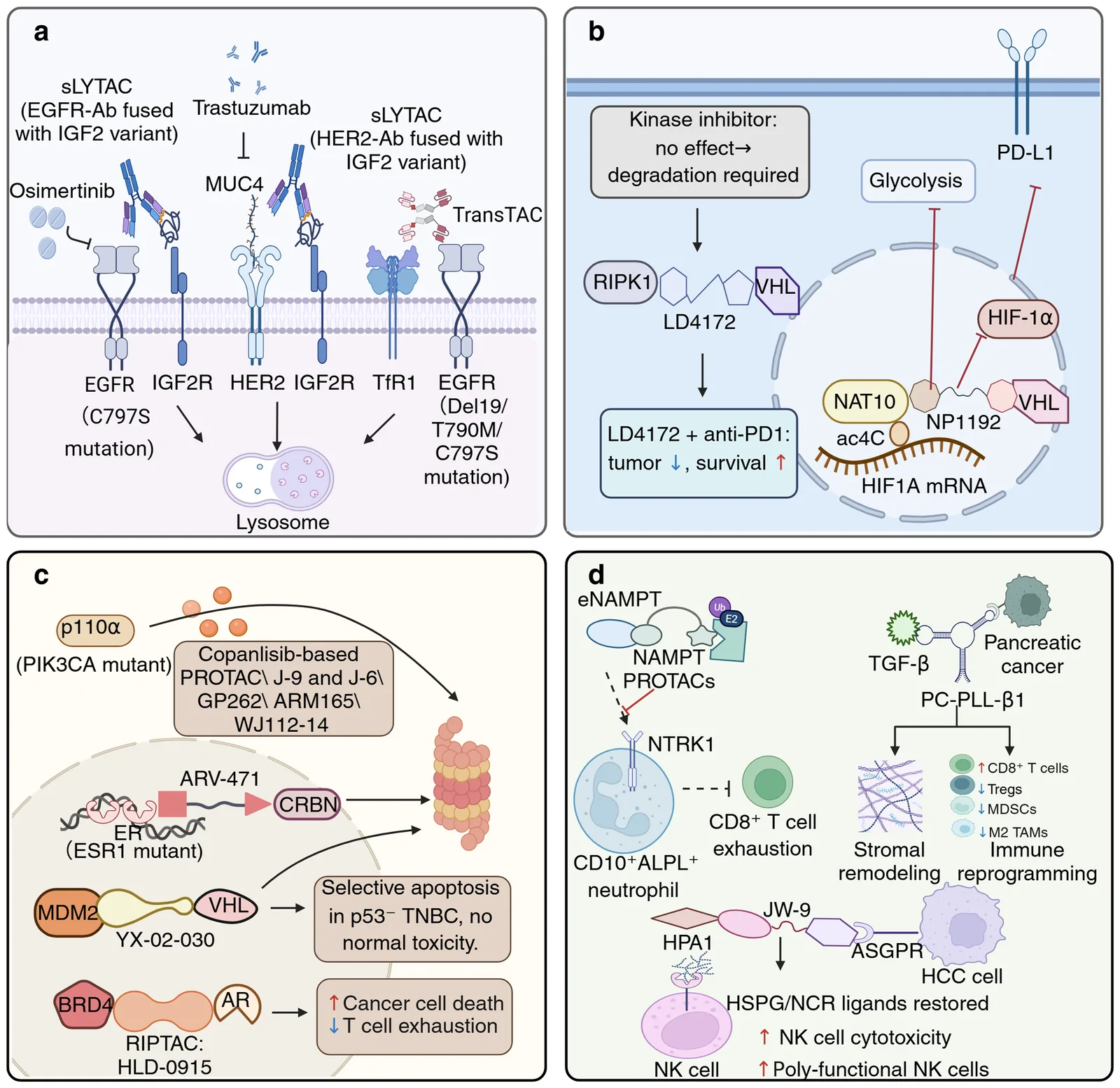
TPD overcomes acquired resistance through degradation of mutated membrane proteins, adaptive signaling hubs, therapeutic targets and metabolic barriers. **(a)** Mutated and upregulated membrane proteins: soluble LYTACs (sLYTACs) fuse therapeutic antibodies with an insulin-like growth factor 2 (IGF2) mutant to redirect complexes to the lysosome via IGF2 receptor (IGF2R), overcoming EGFR C797S kinase inhibitor resistance and HER2/MUC4-masking antibody resistance; transferrin receptor targeting chimeras (TransTACs) leverage transferrin receptor 1 (TfR1) constitutive endocytosis to degrade triple-mutant EGFR (Del19/T790M/C797S). **(b)** Adaptive signaling hubs: the VHL-recruiting PROTAC LD4172 degrades RIPK1, restoring anti-PD-1 efficacy where kinase inhibition fails; NP1192 recruits VHL to degrade NAT10, suppressing N4-acetylcytidine (ac4C)-dependent HIF1A mRNA stabilization and consequent HIF-1α-driven PD-L1 upregulation and glycolysis. **(c)** Targeted therapy resistance: PI3Kα-selective PROTACs restore sensitivity in PIK3CA-mutant cancers; ARV-471 recruits CRBN to degrade mutant ER; the VHL-recruiting PROTAC YX-02–030 degrades MDM2 to stabilize TAp73, upregulating BAX and PUMA for selective apoptosis in p53-deficient TNBC; the RIPTAC HLD-0915 functionally sequesters AR–BRD4, triggering cancer cell death and reducing T cell exhaustion. **(d)** Metabolic and stromal barriers: NAMPT-targeting PROTACs block eNAMPT–NTRK1 signaling to preventCD8^+^ T cell exhaustion; the multivalent aptamer assembly PC-PLL-β1 degrades TGF-β to remodel stroma and reprogramme immunity; the ASGPR-directed LYTAC JW-9 degrades HPA1 to restore heparan sulfate proteoglycan (HSPG)/natural cytotoxicity receptor (NCR) ligands and enhance NK cell cytotoxicity.

Two membrane targets—epidermal growth factor receptor (EGFR) and HER2—illustrate this principle in the context of acquired resistance to kinase inhibitors. In EGFR-mutant lung cancer, the emergence of the cysteine 797 to serine (C797S) mutation abolishes osimertinib binding, while in HER2-positive breast cancer, mucin 4 (MUC4) masking of the trastuzumab epitope limits antibody efficacy. To degrade these recalcitrant membrane proteins, two lysosome-targeting strategies have been developed. Pan and colleagues engineered sLYTACs by fusing an insulin-like growth factor 2 (IGF2) mutant—which binds IGF2 receptor (IGF2R) with high affinity—to therapeutic antibodies, thereby routing antibody–target complexes to the lysosome. For EGFR, an sLYTAC built on nimotuzumab significantly suppressed proliferation in osimertinib-resistant C797S cells. For HER2, an sLYTAC derived from pertuzumab reduced tumor volume by nearly 3.8-fold compared with trastuzumab in the JIMT-1 trastuzumab-resistant xenograft model (18). Separately, Zhang and colleagues developed transferrin receptor targeting chimeras (TransTACs) that leverage high transferrin receptor 1 (TfR1) expression and its rapid constitutive endocytosis to generate bispecific antibodies binding both TfR1 and EGFR, routing mutant EGFR to lysosomal degradation. In an EGFR exon 19 deletion (Del19)/T790M/C797S xenograft model, TransTAC treatment produced potent anti-tumor efficacy: at day 42, four of nine mice showed nearly undetectable tumors, whereas the EGFR Affibody-Fc control exhibited only minimal inhibition (19) (**Figure 5**).

### 4.2 Disrupting adaptive signaling hubs

Adaptive resistance frequently hinges on signaling hubs that reprogram the tumor microenvironment—hubs that TPD can dismantle by degrading the scaffold proteins and enzymes beyond the reach of conventional inhibitors. N-acetyltransferase 10 (NAT10)stabilizes hypoxia-inducible factor 1A (HIF1A) mRNA through N4-acetylcytidine (ac4C) modification, promotes HIF-1α-mediated PD-L1 upregulation, and drives hypoxic glycolysis, collectively shaping an immunosuppressive tumor microenvironment. The conventional inhibitor Remodelin is limited by poor selectivity and susceptibility to resistance. To overcome these limitations, researchers developed NP1192, a PROTAC degrader that recruits the VHL E3 ligase to degrade NAT10. In cervical cancer models, NP1192 achieved nearly 70% NAT10 degradation, inhibited the HIF-1α/PD-L1 axis, reversed hypoxic glycolysis, and demonstrated anti-tumor activity in patient-derived organoids. In anti-PD-L1 resistant models, NP1192 in combination with anti-PD-L1 antibody significantly suppressed tumor growth, increased CD8^+^ effector T cell infiltration, and reduced exhausted T cells, Tregs, and MDSCs, effectively reversing immunotherapy resistance (97).

RIPK1 presents a compelling test case for this principle. A scaffolding protein that mediates both intrinsic and extrinsic resistance to immune checkpoint blockade, receptor-interacting protein kinase 1 (RIPK1) has remained undruggable by conventional inhibitors due to its poorly defined intermediate domain. Yu et al. developed LD4172, a PROTAC degrader that selectively eliminates RIPK1 via VHL-mediated proteasomal degradation. In anti-PD1-resistant B16F10 melanoma and MC38 colon cancer models, LD4172 combined with anti-PD1 significantly suppressed tumor growth (p < 0.001) and extended survival, whereas a RIPK1 kinase inhibitor showed no such effect—confirming that degradation, not kinase inhibition, is required to overcome resistance (98) (**Figure 5**).

### 4.3 Reversing targeted therapy resistance

This ability of TPD to bypass inhibitor resistance extends to small-molecule targeted therapies, where acquired mutations frequently undermine conventional agents. In estrogen receptor (ER) -positive advanced breast cancer, endocrine therapy frequently encounters resistance driven by activating estrogen receptor 1 (ESR1) mutations. The standard agent fulvestrant achieves only 50%–60% ER degradation in the clinic, falling short of therapeutic needs. To address this challenge, Arvinas developed vepdegestrant (ARV-471), an oral PROTAC degrader that induces the formation of an ER: PROTAC: CRBN ternary complex, leading to direct ER ubiquitination and subsequent proteasomal degradation (99). In the phase IIItrial (NCT05654623), vepdegestrant significantly improved median progression-free survival (PFS) over fulvestrant in ESR1-mutated advanced breast cancer (5.0 vs 2.1 months) (5). Another oral SERD, camizestrant, also showed superior PFS over fulvestrant in the phase II SERENA-2 trial (NCT04214288) (7.2–7.7 vs 3.7 months; HR 0.59–0.64) (100). In the phase III SERENA-6 trial (NCT04964934), patients with emerging ESR1 mutations on first-line aromatase inhibitor plus cyclin-dependent kinase 4 and 6 (CDK4/6) inhibitor without progression were switched to camizestrant plus the same CDK4/6 inhibitor, which significantly improved median PFS over continuing the original regimen (16.0 vs 9.2 months; HR = 0.44) and delayed quality-of-life deterioration. These findings support the clinical value of next-generation oral SERDs in overcoming endocrine resistance driven by ESR1 mutations (101).

Beyond endocrine resistance, dysregulation of the phosphoinositide 3-kinase (PI3K)–protein kinase B (AKT)–mechanistic target of rapamycin (mTOR) axis represents another major driver of targeted therapy failure. As phosphatidylinositol-4,5-bisphosphate 3-kinase catalytic subunit alpha (PIK3CA) mutations or phosphatase and tensin homolog (PTEN) loss hyperactivate this pathway, it drives resistance to agents including lapatinib across multiple cancers (102). To address this, several TPD strategies have been developed. Inavolisib, an adenosine triphosphate (ATP) -competitive inhibitor, also promotes the selective degradation of mutant p110α via the ubiquitin-proteasome system (103). In a Phase I trial, Inavolisib demonstrated a manageable safety profile at the maximum tolerated dose in patients with PIK3CA-mutant HR+/HER2− advanced breast cancer, with an objective response rate of 26% (104). In addition, several PROTACs have been developed as follows. The Copanlisib-based PROTAC restored drug sensitivity in lapatinib-resistant HER2+ breast cancer xenografts and patient-derived organoids (102). Based on the TGX221 inhibitor, the PI3Kβ-selective PROTACs J-9 and J-6 respectively showed strong synergy with adriamycin and cisplatin in multidrug-resistant xenografts/cells (105). The dual-target PROTAC GP262 degrades p110α and mTOR, achieving 79.2% tumor growth inhibition (TGI) at 25 mg/kg in MDA-MB-231xenografts (106). ARM165, based on the AZ2 inhibitor, reduced leukemia burden in acute myeloidleukaemia (AML) mouse models and patient-derived xenograft(PDX), and synergized with venetoclax (107). Based on a 4-methylquinazoline scaffold, Compound 12 achieved a TGI rate of 61.8% in DOHH2 lymphoma, surpassing positive controls ibrutinib and idelalisib (108). The PROTAC WJ112-14, based on the pan-PI3K module PQR514, selectively degraded PI3Kα and outperformed taselisib in PIK3CA-mutant cell lines (109). In summary, PI3K-targeting TPD strategies effectively overcome resistance to conventional inhibitors.

KRAS illustrates both the promise and the difficulty of extending TPD to non-enzymatic scaffolding proteins (110, 111). Although covalent small-molecule inhibitors such as sotorasib—a small molecule that selectively and irreversibly targets KRASG12C—have recently achieved clinical breakthroughs, with surprising advances in direct KRAS-targeting drugs, especially KRAS(G12C) inhibitors (112, 113), PROTAC-mediated degradation of KRAS has now been demonstrated across oncogenic alleles: LC-2, the first PROTAC capable of degrading endogenous KRASG12C, uses an MRTX849-derived covalent warhead to recruit VHL and suppress MAPK signaling (110), and the CRBN-recruiting degrader RP03707 achieves potent, selective KRASG12D degradation with efficacy in xenograft models (111). Nevertheless, KRAS presents structural challenges rooted in its biology. It functions as a signaling hub in multiple cellular pathways regulating proliferation, differentiation, growth, metabolism and migration (114), and its effector-binding regions comprise two shallow binding pockets, switch I and switch II, whose conformations change dramatically between the GDP- and GTP-bound states (110). The smooth surface and lack of deep binding pockets have long marked KRAS as undruggable (114, 115), and mutant KRAS has remained a challenging therapeutic target given the scarcity of traditional druggable pockets on its surface (110). Productive degradation must overcome these conformational constraints: studies of GTP-loaded KRAS(on) degraders show that degradation potency is governed by ternary-complex affinity, stability and cooperativity, and ACBI4, which forms a highly stable and cooperative ternary complex with VHL and GTP-bound KRAS, potently degrades KRAS G12R (116). Moreover, KRAS activates downstream effectors, most notably the RAF kinase, and the PI3K/AKT/mTOR pathway is another key route activated by RAS (115). Because degradation eliminates scaffolding functions that are not typically attenuated by traditional small-molecule inhibitors (110)—consistent with the principle that single-agent active-site inhibitors are sometimes unable to eliminate the scaffolding (non-catalytic) functions of oncogenic kinases (117)—complete degradation, rather than catalytic inhibition, is mechanistically attractive for eliminating these effector-mediated functions. The choice of E3 ligase (CRBN, VHL, DCAF1 or KLHDC2), using a KRAS-G12D binder derived from MRTX1133, critically determines degradation potency and selectivity (118). Efficient assembly of a productive ternary complex nevertheless remains the central challenge for KRAS, and these considerations underscore that non-enzymatic scaffolding proteins present hurdles to TPD beyond target identity, motivating continued efforts in ligand engineering and E3 ligase diversification.

Beyond kinase signaling, TPD strategies also address resistance driven by nuclear hormone receptors, as exemplified by the androgen receptor in prostate cancer. The androgen receptor (AR) is a key intracellular immunomodulatory target. AR directly suppresses T cell IFNγ production and negatively regulates PD-L1 expression, while driving CD8^+^ T cell exhaustion via epigenetic and transcriptional programming (119). In tumor-associated macrophages, AR is activated through the APOE-TREM2 axis and upregulates immunosuppressive factors such as IL-10 and TGF-β (120). Although AR signaling inhibitors are the standard of care for prostate cancer, nearly all patients with metastatic castration-resistant prostate cancer (mCRPC) eventually develop resistance, driven by heterogeneous bypass mechanisms including AR gene amplification and AR mutations, leaving a significant unmet clinical need (121). To overcome this resistance, HLD-0915—a Regulated Induced Proximity Targeting Chimera (RIPTAC) distinct from PROTAC degraders—forms a trimeric complex with AR and the essential survival protein bromodomain-containing protein 4 (BRD4), functionally sequestering BRD4 without inducing its degradation (122). This AR-dependent BRD4 inactivation selectively triggers cell death in prostate tumors, including treatment-resistant models, while sparing AR-negative cells. HLD-0915 has demonstrated oral efficacy with significant tumor regression and prostate-specific antigen (PSA) reduction in preclinical studies, along with a favorable therapeutic index, and is currently in Phase I evaluation (NCT06800313). This strategy offers a novel approach to overcome AR-targeted drug resistance (123).

TPD further exploits vulnerabilities arising from tumor suppressor inactivation, as illustrated by mouse double minute 2 (MDM2) dependency in p53-mutant cancers. In p53-inactivated triple-negative breast cancer, tumors exhibit intrinsic resistance to conventional chemotherapy and MDM2 small-molecule inhibitors due to p53 mutation or deletion. Adams and colleagues discovered an unexpected survival dependency of these tumors on MDM2 and developed a targeted MDM2 PROTAC degrader, YX-02-030. This molecule efficiently degrades MDM2 by recruiting the VHL E3 ligase, thereby stabilizing and activating transactivation domain-containing p73 (TAp73), upregulating pro-apoptotic genes such as BCL2-associated X (BAX) andp53 upregulated modulator of apoptosis (PUMA), and selectively inducing apoptosis in p53-mutant or p53-deleted TNBC cells without toxicity to normal cells. This PROTAC demonstrated significant anti-tumor activity in patient-derived relapsed tumor samples and xenograft models. This study reveals MDM2 as a targetable vulnerability in p53-inactivated tumors and provides a new strategy to overcome therapy resistance associated with p53 inactivation (124) (**Figure 5**).

### 4.4 Degrading metabolic and stromal barriers

Resistance to therapy also originates from metabolic and stromal components of the tumor microenvironment, which have proven amenable to targeted degradation. Anti-PD-1 therapy, standard for advanced HCC, often fails owing to resistance. tumor-secreted extracellular nicotinamide phosphoribosyltransferase (eNAMPT) reprograms CD10^+^ALPL^+^ intratumoral neutrophils via neurotrophic tyrosine receptor kinase 1 (NTRK1), driving CD8^+^ T cell exhaustion and anti-PD-1 resistance (125). Secreted eNAMPT acts as a pro-inflammatory cytokine, promoting immune evasion via TLR4/NF--κB activation (126). Conventional inhibitors (e.g., FK866, CHS-828) inhibit only intracellular NAMPT (iNAMPT), sparing eNAMPT, and failed clinically due totoxicity (127). This has spurredthe development of NAMPT--targeting PROTACs. Cheng et al. developed B4, a theranostic PROTAC derived from the fluorescent NAMPT inhibitor A1, achieving 73% tumor inhibition in xenograft models, whereas the parental inhibitor A1 caused universal lethality due to severe toxicity (128). Bi et al. developed the VHL--recruiting degrader B3, achieving sub-nanomolar degradation in A2780 cells and 88.1% tumor inhibition in an A2780 xenograft model, outperforming FK866 and the parental MS0 without overt toxicity (129). Lu et al. developed the VHL--recruiting degrader LYP-8, which achieved 46% tumor inhibition in the CT-26 syngeneic model (vs. 16% for FK866) (127). Zhu et al. developed first--generation CRBN--based degraders SIAIS630120/630121 and a second--generation molecule 632005, which eliminates the hook effect via a bicyclic scaffold, extends half-life to 5.98 h, reduces clearance 2- to 3-fold,and establishes nicotinamide phosphoribosyltransferase (NAMPT) deficiency as a sensitivity marker for nicotinic acid--mediated selective protection (126, 130).

Whereas eNAMPT exemplifies a metabolically active secreted factor driving immune exhaustion, TGF-β illustrates how a pleiotropic cytokine concurrently enforces immune suppression and stromal remodeling. TGF-β directly promotes the expansion and function of Tregs, potently suppressing the activity of effector T cells. Within the innate immune system, it exerts dual regulatory effects: on one hand, it inhibits the function of NK cells, while on the other hand, it modulates the activity of macrophages, DCs, and granulocytes (131). M7824, a PD--L1/TGF--β dual--targeting fusion protein, failed Phase III trials in NSCLC and TNBC, possibly because its TGF--βRII domain inadequately captured TGF--β ligands, leading to incomplete signaling suppression or paradoxical pathway activation (132). To overcome the limited tumor-targeting and systemic toxicity of TGF--β blockade, Liu et al. developed a multivalent aptamer assembly, PC--PLL--β1, which was constructed by self--assembly of a pancreatic cancer--specific aptamer, a TGF-β--targeted aptamer, and poly--L--lysine, and enables tumor-specific lysosomal degradation of TGF--β1. In a gemcitabine--resistant Panc-1/PSC model, PC--PLL--β1 plus gemcitabine elevated tumor inhibition from 9.5% to 61.6% and halved intratumoral collagen, underscoring its ability to reverse chemoresistance via stromal remodeling. In a syngeneic Panc02 model, the assembly combined with ovalbumin achieved 65.8% tumor inhibition versus 13.1% with ovalbumin alone, markedly increasing CD8^+^ T cell infiltration while reducing regulatory T cells, M2--like macrophages, and myeloid--derived suppressor cells, thereby effectively counteracting immunotherapy resistance (133).

Local degradation within the tumor microenvironment is feasible and has been demonstrated, but so far only by tumor-compartment-restricted designs. A multivalent aptamer assembly (PC-PLL-β1) drags extracellular proteins such as TGF-β1 to lysosomal degradation with high tumor specificity, exhibits highly tumor-specific accumulation and prolonged retention *in vivo*, and achieves efficient TGF-β inhibition, stromal remodeling and reversal of immunosuppressive-cell polarization within the tumor microenvironment (133). Likewise, a self-assembling LYTAC nanoplatform (NanoCLY) designed for tumor-microenvironment-responsive degradation of CTGF achieved complete degradation of this soluble mediator, downregulating TGF-β signaling and disrupting CTGF-IL-6 crosstalk within the tumor microenvironment (58). Nevertheless, because soluble factors such as TGF-β diffuse systemically, degradation confined to the tumor compartment cannot address the systemic pool; the limited spatial reach of current soluble-protein degraders is therefore a genuine limitation of LYTAC-based strategies for secreted targets. Improving tumor retention—extending the tumor-specific accumulation and prolonged retention demonstrated for PC-PLL-β1 (133)—and exploiting ligand-receptor pairs with restricted expression are among the strategies being explored, although small-molecule LYTACs capable of degrading soluble TGF-β have not yet been reported and current evidence relies on macromolecular platforms.

A more localized stromal barrier operates in hepatocellular carcinoma, where extracellular heparanase disrupts NK cell recognition independent of cytokine networks. HCC-derived heparanase 1 (HPA1) cleaves heparan sulfate side chains on the tumor surface, thereby depleting natural cytotoxicity receptor (NCR) ligands such as heparan sulfate proteoglycans (HSPGs) and enabling immune evasion. However, conventional HPA1 inhibitors only block enzymatic activity with limited clinical efficacy. To address this challenge, Li and colleagues developed a nonproteinogenic compound-conjugated LYTAC degrader, JW-9. This molecule conjugates the small-molecule HPA1 inhibitor OGT2115 to a tri-GalNAc ligand for ASGPR, enabling selective degradation of extracellular HPA1 via ASGPR-mediated endolysosomal trafficking. In HepG2 cells, JW-9 degraded HPA1 in a concentration-dependent manner and restored membrane HSPG/NCR ligand abundance, whereas OGT2115 alone had no such effect. Functionally, JW-9 pretreatment significantly enhanced NK cell cytotoxicity against HepG2 cells, upregulated NK activating receptors, and increased the proportion of poly-functional effector NK cells, outperforming OGT2115. Collectively, this heparanase-specific LYTAC degrader effectively reverses immune resistance and potentiates NK cell anti-tumor function (134) (**Table 1**, **Figure 5**).

**Table 1:** Representative TPD agents: modality, stage and key efficacy data.

Agent	Target	Modality/E3 or receptor	Stage	Key efficacy data	Trial identifier/reference
CXCL12–Atz (KineTAC)	PD-L1	Bispecific KineTAC/CXCR7	Preclinical	~70% PD-L1 degradation in MDA-MB-231 cells; restored T-cell cytotoxicity; reduced xenograft growth	(49)
PD-LYSO	PD-L1	Chimeric peptide (HIP1R lysosomal sorting signal)	Preclinical	Routes PD-L1 to lysosomal degradation; superior anti-tumor efficacy versus conventional antibodies	(50)
P22	PD-L1	PROTAC-like bifunctional molecule (lysosome-dependent)	Preclinical	Restores T-cell immune responses; superior to Keytruda	(51)
BMS-L1-RGD (IFLD)	PD-L1	IFLD/integrin	*In vitro*	Highly efficient PD-L1 degradation	(20)
AC-1 (AbTAC)	PD-L1	AbTAC/RNF43	*In vitro*	Induces lysosomal degradation of PD-L1	(8)
CD71 LYTAC	CD71	LYTAC/CI-M6PR	*In vitro*	>80% CD71 degradation in Jurkat T cells; blocks transferrin uptake	(7)
Nanosphere-AntiCD24	CD24	Nano-LYTAC/ASGPR	Preclinical	Enhances macrophage phagocytosis; better *in vivo* stability than free antibody; no observable liver toxicity	(22)
FRTAC	CD47	FRTAC/folate receptor	*In vitro*	Degrades CD47 via folate-receptor-mediated endocytosis	(21)
MD13	MIF	PROTAC/CRBN	*In vitro*	DC50 ≈ 100 nM (A549); ERK/MAPK inactivation; G2/M arrest; anti-proliferative effect	(57)
NanoCLY (CL8-M6P3)	CTGF	Peptide LYTAC/CI-M6PR	Preclinical	TGI 59.62% in TNBC; synergy with paclitaxel; suppressed lung and bone metastasis; superior to FG-3019	(58)
Nanobody–enzyme fusions (anti-HER2/Trop2-VHH–DPP4/SubA)	sICOSL/sGRP78	Nanobody–enzyme conjugates (extracellular proteolysis)	Preclinical	Specifically clears soluble factors in the TME; reverses immunosuppression; enhances chemotherapy efficacy	(59, 61)
PF	FOXP3	PROTAC/CRBN	Preclinical	Depletes Tregs; CD8+ T-cell-dependent anti-tumor activity; single-agent and anti-PD-1 combination efficacy in the RENCA model	(76)
KT-333	STAT3	PROTAC/VHL	Phase I	3 CR (cHL, NK-cell lymphoma), 4 PR (CTCL); >90% STAT3 degradation in blood and tumor tissue; IFN-γ gene signature	NCT05225584 (6)
Iberdomide	IKZF1/IKZF3	Molecular glue/CRBN	Phase I/II	ORR 26–32% in heavily pretreated MM; overcomes IMiD resistance	NCT02773030 (90)
KT-413	IRAK4 + IKZF1/3	PROTAC/CRBN	Phase I	Preclinical studies completed; Phase I in B-cell lymphoma	NCT05233033 (92)
ARV-393	BCL6	PROTAC	Phase I	Phase I in relapsed/refractory NHL	NCT06393738
DT2216	BCL-XL	PROTAC/VHL	Phase I completed; Phase I/II ongoing	Selective depletion of TI-Tregs with platelet sparing; T-cell activation	NCT04886622; NCT06620302 (96)
sLYTAC (nimotuzumab-sLYTAC)	EGFR (C797S)	sLYTAC/IGF2R	*In vitro*	Suppresses proliferation of osimertinib-resistant C797S cells	(18)
sLYTAC (pertuzumab-sLYTAC)	HER2 (MUC4 masking)	sLYTAC/IGF2R	Preclinical	~3.8-fold reduction in tumor volume versus trastuzumab in JIMT-1 trastuzumab-resistant xenografts	(18)
TransTAC	Triple-mutant EGFR (Del19/T790M/C797S)	TransTAC/TfR1	Preclinical	4/9 mice with nearly undetectable tumors at day 42; Affibody-Fc control showed only minimal inhibition	(19)
NP1192	NAT10	PROTAC/VHL	Preclinical	~70% NAT10 degradation; HIF-1α/PD-L1 axis inhibition; significant tumor suppression when combined with anti-PD-L1 in resistant models	(97)
LD4172	RIPK1	PROTAC/VHL	Preclinical	Tumor suppression with anti-PD-1 in B16F10/MC38 (p<0.001) with extended survival; kinase inhibitor ineffective	(98)
Vepdegestrant (ARV-471)	ERα	PROTAC/CRBN	Phase III (oral)	mPFS 5.0 vs 2.1 months in ESR1-mutant advanced breast cancer (vs fulvestrant)	NCT05654623 (5)
Camizestrant	ERα	Oral SERD	Phase II/III (oral)	SERENA-2: mPFS 7.2–7.7 vs 3.7 months; SERENA-6: 16.0 vs 9.2 months	NCT04214288; NCT04964934 (100)
Inavolisib	Mutant PI3Kα	Inhibitor + degrader	Phase I (oral)	ORR 26% in PIK3CA-mutant HR+/HER2− breast cancer; manageable safety profile at the maximum tolerated dose	NCT03006172 (104)
Copanlisib-PROTAC	PI3K	PROTAC	Preclinical	Restores drug sensitivity in lapatinib-resistant HER2+ breast cancer xenografts and PDOs	(102)
J-9/J-6	PI3Kβ	PROTAC	Preclinical	Strong synergy with adriamycin and cisplatin in multidrug-resistant models	(105)
GP262	p110α + mTOR	Dual-target PROTAC	Preclinical	TGI 79.2% at 25 mg/kg in MDA-MB-231 xenografts	(106)
ARM165	PI3K	PROTAC	Preclinical	Reduces AML burden; synergizes with venetoclax	(107)
Compound 12	PI3K	PROTAC	Preclinical	TGI 61.8% in DOHH2 lymphoma; superior to ibrutinib and idelalisib	(108)
WJ112-14	PI3Kα	PROTAC	*In vitro*	Selectively degrades PI3Kα; outperforms taselisib in PIK3CA-mutant cells	(109)
HLD-0915	AR–BRD4 (RIPTAC)	Induced proximity	Phase I (oral)	Oral efficacy; tumor regression; PSA reduction in mCRPC models; favorable therapeutic index	NCT06800313 (122, 123)
YX-02-030	MDM2	PROTAC/VHL	Preclinical	Stabilizes TAp73 and upregulates BAX/PUMA; selective apoptosis in p53-mutant/deleted TNBC; activity in PDX and xenografts	(124)
B4	NAMPT (eNAMPT)	Theranostic PROTAC	Preclinical	TGI 73% in xenografts; parental inhibitor A1 caused universal lethality	(128)
B3	NAMPT	PROTAC/VHL	Preclinical	Sub-nanomolar degradation in A2780 cells; TGI 88.1% in A2780 xenografts	(129)
LYP-8	NAMPT	PROTAC/VHL	Preclinical	TGI 46% in the CT-26 syngeneic model (vs 16% for FK866)	(127)
SIAIS630120/632005	NAMPT	PROTAC/CRBN	Preclinical	632005 abolishes the hook effect; half-life 5.98 h; 2- to 3-fold reduced clearance	(126, 130)
PC-PLL-β1	TGF-β1	Multivalent aptamer assembly (lysosomal)	Preclinical	TGI 9.5%→61.6% with gemcitabine in resistant Panc-1/PSC; 65.8% vs 13.1% with ovalbumin in Panc02; halved intratumoral collagen	(133)
JW-9	Extracellular HPA1	LYTAC/ASGPR	Preclinical	Restores HSPG/NCR ligands; enhances NK cell cytotoxicity	(134)

CR, complete response; PR, partial response; ORR, overall response rate; TGI, tumor growth inhibition; mPFS, median progression-free survival; TI-Treg, tumor-infiltrating regulatory T cell; MM, multiple myeloma; NHL, non-Hodgkin lymphoma; mCRPC, metastatic castration-resistant prostate cancer; TNBC, triple-negative breast cancer; PDO, patient-derived organoid; PDX, patient-derived xenograft; AML, acute myeloid leukaemia; MDR, multidrug resistant; PSA, prostate-specific antigen; SERD, selective estrogen receptor degrader; TME, tumor microenvironment; KineTAC, kinetokine-targeting chimera; AbTAC, antibody-based PROTAC; IFLD, integrin-facilitated lysosomal degradation; FRTAC, folate receptor-targeting chimera; sLYTAC, soluble LYTAC; TransTAC, transferrin receptor-targeting chimera; CI-M6PR, cation-independent mannose 6-phosphate receptor; ASGPR, asialoglycoprotein receptor.

## 5 Translational challenges

**Figure 6:**
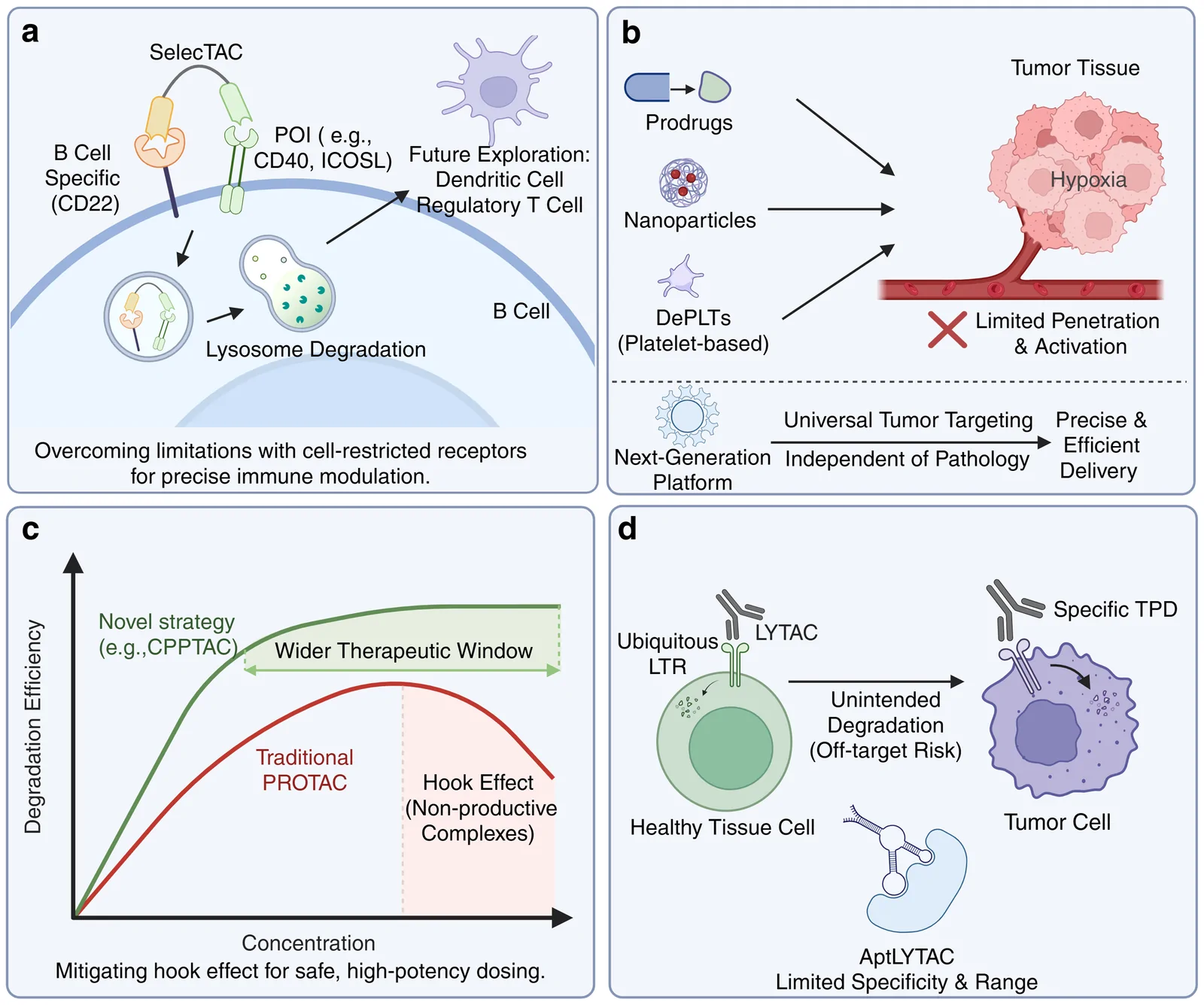
Overcoming on-target toxicity, hook effect constraints and delivery limitations to advance TPD translation. **(a)** SelecTACs leverage lineage-restricted receptors (e.g., CD22 on B cells) to deliver TPD cargos selectively to immune subsets, enabling precise lysosomal degradation of immunomodulatory proteins (e.g., CD40, ICOS) while sparing bystander cells. Future applications include targeting dendritic cells and regulatory T cells for tailored immune regulation. **(b)** Conventional delivery platforms (prodrugs, nanoparticles, platelet-based DePLTs) face limitations in tissue penetration and activation in hypoxic solid tumors, highlighting the need for next-generation universal targeting strategies that function independently of pathological context. **(c)** Traditional PROTACs exhibit reduced degradation efficiency at high concentrations due to non-productive binary complexes, narrowing the therapeutic window. Novel modalities such as CPPTACs mitigate this effect, enabling safe, high-potency dosing across a wider concentration range. **(d)** LYTACs relying on ubiquitously expressed lysosomal targeting receptors (LTR) carry risks of unintended degradation in healthy tissues. TPD platforms with tumor-specific targeting (e.g., AptLYTACs, selective degraders) minimize off-target activity by restricting activity to the tumor microenvironment.

Among the barriers to translating these advances into clinical practice, off-target effects pose a particularly significant safety concern. In the application of TPD technologies to cancer immunotherapy, such effects primarily manifest as unintended protein degradation and functional perturbation from non-degradative off-target binding—a risk exemplified by LYTAC, whose reliance on widely expressed LTR may lead to non-specific endocytosis and off--target degradation (23). The risk of on-target toxicity in normal tissues is not merely theoretical—it is already observable in the very targets that make TPD attractive for immunotherapy. PD-L1, for instance, is upregulated on tumor cells but is also expressed on activated T cells as an intrinsic negative feedback regulator; degrading it indiscriminately could, in principle, impair the effector functions of tumor-infiltrating lymphocytes while depleting the tumor-associated pool. Similarly, CD47 is widely expressed not only on tumor cells but also on erythrocytes and platelets, and systemic CD47 degradation has been a major cause of hematological toxicity in antibody programs. The BCL-XL degrader DT2216offers an instructive counterexample: by recruiting VHL, an E3 ligase with low expression in platelets, it achieves selective depletion of tumor-infiltrating Tregs while sparing circulating platelets. These examples illustrate that the safety profile of TPD in normal immune and hematopoietic compartments is not determined by target choice alone, but critically by degrader design—particularly E3 ligase selection and tumor-restricted delivery—a point that remains underappreciated in the current literature. Although aptamer-based lysosome-targeting chimeras (AptLYTACs) enable the simultaneous degradation of two proteins, their targeting specificity remains relatively weak and the spectrum of addressable proteins is narrow (135) (**Figure 6**).

For degraders recruiting cereblon (CRBN) as the E3 ubiquitin ligase, simple potency screening cannot resolve neosubstrate-related safety risks: CRBN ligands induce ternary complexes that recruit additional neosubstrates, and degradation of such proteins may raise safety concerns when it falls outside the intended pharmacological profile (41). Two classes of strategies improve degrader selectivity in this setting. First, multiparameter optimization (MPO) scoring: Szewczyk et al. analyzed a glutarimide CELMoD library to identify interpretable chemical features correlated with neosubstrate selectivity, and developed a simple multiparameter optimization function using only three parameters to predict whether molecules will have undesired neosubstrate activity (136). Second, structure-guided chemical modification of the CRBN-binding ligand: in the KT-474 campaign, removal of an additional hydrogen-bond donor maintained CRBN affinity and improved passive permeability, within an effort explicitly aimed at novel CRBN binders devoid of IMiD (IKZF1/3) neosubstrate degradation (137), and dihydrouracil-based scaffold replacement similarly mitigates IMiD-associated safety liabilities (41). Beyond these ligand-centric strategies, allosteric modulation of CRBN itself—exemplified by SB-405483, which alters the neosubstrate degradation profiles of orthosteric ligands (138)—represents a mechanistically distinct route to reshape substrate selectivity. Together, these approaches provide complementary, safety-oriented optimization strategies for CRBN-recruiting degraders (41, 136, 138). Importantly, plastic interprotein contacts within ligand-induced ternary complexes confer selectivity for ligand-induced protein degradation, providing a conceptual framework for the development of heterobifunctional degraders (139); conversely, structural knowledge of E3 ligase substrate-recognition surfaces is guiding the application of structure-based design approaches to the development of new and existing degraders as chemical tools and therapeutics (140).

The “limited specificity and range” of aptamer-based lysosome-targeting chimeras (AptLYTACs) are constrained by multiple factors (24, 28). Nucleic acid aptamers, often termed ‘chemical antibodies’, are functionally comparable to traditional antibodies but offer several advantages, including versatile chemical modification and high stability (28); nevertheless, their clinical translation has been delayed by inherent physicochemical characteristics, including susceptibility to nuclease degradation and rapid renal clearance (28). Beyond these pharmacokinetic constraints, the tissue selectivity of AptLYTACs is additionally determined by the lysosome-shuttling receptor used, as exemplified by the liver-cell-specific degradation achieved when aptamers are conjugated to tri-GalNAc (24). Chemical modification—a hallmark advantage of aptamers—is a central strategy for improving their stability (28). At the level of responsive linker design, acid-degradable linkers have been developed to release their cargo in mildly acidic endolysosomal compartments: the azido-acetal linker, for example, has a pH 6.0 hydrolysis half-life (t1/2) of 14.8 min and a hydrolysis t1/2 of 21 days at pH 7.4 (141). This kinetic profile enables the linker to remain stable at physiological pH and to become activated upon reaching acidic endolysosomal compartments (141). Furthermore, tumor acidosis is not merely a bystander feature of accelerated metabolism: substantial evidence indicates that acidosis participates in cancer progression through stimulation of autophagy and immunosuppression, providing a physiological rationale for pH-responsive prodrug design (142). In parallel, enzyme-cleavable linkers activated by cathepsin B or β-galactosidase have been developed for endolysosomal release, providing further strategies to enhance tissue specificity (141). In principle, linkers responding to the mildly acidic tumor extracellular microenvironment (rather than acting only after internalization into endolysosomes) could reduce systemic exposure; however, the azido-acetal kinetics described above correspond to endolysosomal pH conditions (approximately 5.0–6.0) (141), so exploiting the milder acidity of the tumor extracellular microenvironment would require dedicated linker design and validation. These design principles, already validated in drug delivery, provide a translatable framework for optimizing next-generation AptLYTACs; however, the *in vivo* stability, cleavage kinetics, and translational feasibility of such linker systems require systematic evaluation in relevant preclinical models (141).

Even when target specificity is achieved, the intrinsic pharmacology of degraders introduces a separate hurdle. When optimizing the clinical applicability of targeted protein degraders, the hook effect represents another key pharmacological challenge that constrains the therapeutic window. An ideal degrader should simultaneously exhibit rapid onset, high potency, a high maximal degradation level (Dmax), and sustained target protein clearance (84). However, traditional PROTAC--type molecules often suffer from reduced degradation efficiency at high concentrations due to the “hook effect”—where an excess of degrader molecules preferentially forms unproductive binary complexes with either the target protein or the E3 ligase, thereby impeding the assembly of functional ternary complexes, this phenomenon substantially narrows their safe and effective dosing range (143). To overcome this mechanistic limitation, novel degradation strategies have been developed, such as CPPTAC, which fuses a target protein ligand with a cell--penetrating peptide and exploits its interaction with cell--surface glycans to mediate endocytosis; preliminary studies indicate that this mechanism can mitigate the hook effect across a wider concentration range (25). Consequently, simultaneously evading the hook effect while co--optimizing degradation efficiency and tumor-targeting specificity has become a critical challenge for advancing the clinical translation of TPD (**Figure 6**).

DC50, the concentration of 50% protein degradation (144), and Dmax, the maximum degradation efficacy, are the commonly used metrics for reporting degradation efficiency (145). Importantly, however, these metrics are time-dependent, which limits direct comparability across studies that use different measurement time points (145); consequently, DC50 and Dmax values reported under different assay conditions are frequently not directly comparable, which can distort cross-study benchmarking of clinical candidates (145). To ensure consistency across the clinical data cited in this Review, we recommend that degradation metrics be reported together with (i) the exact assay format (endogenous versus ectopic target, cell line and passage number), (ii) the time point of measurement relative to treatment, (iii) the method of protein quantification, and (iv) the full dose–response curves from which DC50 and Dmax are derived, rather than single-point values (145). Adoption of such standardized reporting—ideally complemented by degradation kinetics, including the rate and duration of target degradation (84)—will be essential for the quantitative benchmarking and clinical translation of targeted protein degraders. Chemogenetic tools that enable acute, time-resolved degradation of single proteins—most notably the dTAG system—confer kinetic resolution to biological investigation and provide target validation in the context of drug discovery (146), underscoring the value of reporting time-dependent degradation profiles.

In addition to overcoming resistance to conventional inhibitors, TPD must contend with resistance mechanisms inherent to the degradation machinery itself (147). These mechanisms can be grouped into several classes: (i) E3 ligase loss or mutational inactivation; (ii) drug-efflux-mediated clearance; (iii) target-protein mutation; (iv) deubiquitinase upregulation; and (v) alterations in proteasome/UPS components, with compensatory autophagy representing an additional, proteasome-independent dimension. One well-characterized mechanism is the downregulation or mutational inactivation of E3 ligases—most notably CRBN and VHL—which directly abrogates PROTAC-mediated ubiquitination (148). Zhang et al. demonstrated that chronic exposure to VHL-based BET-PROTACs led to CUL2 loss via multiple genomic alterations—including frameshift mutation, exon skipping, and large-scale deletion—which abolished the function of the VHL–CRL complex and conferred resistance; similarly, resistance to CRBN-based BET-PROTACs was attributed to CRBN gene deletion (148). Notably, cells resistant to VHL-based PROTACs remained sensitive to CRBN-based PROTACs, and vice versa, indicating that resistance is E3 ligase-specific and that switching E3 ligase engagement may represent a viable strategy to overcome ligase-mediated resistance (148). Beyond E3 ligase loss, acquired resistance can also arise through upregulation of drug efflux pumps: expression of ATP-binding cassette (ABC) transporters, especially multidrug resistance protein 1 (MDR1, also known as P-glycoprotein or P-gp), can confer resistance to cytotoxic and targeted chemotherapy (149), and chronic exposure to BET protein or CDK9 degraders induced MDR1 expression via gene amplification and/or promoter hypomethylation, leading to reduced intracellular PROTAC accumulation and a marked rightward shift in degradation DC50 values—a resistance mechanism that could be reversed by MDR1 inhibition (150). In addition, mutations in the target protein itself can confer resistance to degraders in the clinic (151): Sievers et al. sequenced serial CLL samples from patients enrolled in the phase I trials of the BTK degraders zelebrudomide and bexobrutideg and observed recurrent expansion of preexisting BTK A428D mutations at relapse; a crystal structure of BTK A428D revealed that the mutant aspartate clashes with the adenine ring of ATP and the adenine-mimetic moiety of BTK inhibitors and degraders (151). Importantly, unbiased cellular screens have shown that cancer cell lines raised to resist degraders manifest a degrader-specific mechanism of resistance resulting from the loss of components of the ubiquitin-proteasome system (147). Deubiquitinases (DUBs) add another layer of resistance: the suitability of DUBs as substrates for targeted protein degradation is now being systematically assessed, with some DUBs readily degradable via the ubiquitin-proteasome pathway and others resisting turnover through auto-deubiquitylation or as poor proteasome substrates (152); consistent with this, USP28 is markedly upregulated in various solid tumors and promotes proliferation and therapeutic resistance by stabilizing oncoproteins such as c-Myc, STAT3 and SOX9 (153). Compensatory autophagy has been proposed as a further dimension of resistance. In proteasome-inhibition models—the context in which proteasome-to-autophagy switching is best established—proteasome inhibition upregulates BAG3, which significantly contributes to the compensatory activation of autophagy and to the degradation of the (poly)ubiquitinated proteins (154); loss of the UPS shuttle-protease DDI2 triggers CCN1-driven compensatory autophagy (155); and TRIM24 plays a pivotal regulatory role in proteasome-autophagy crosstalk in bortezomib-resistant mantle cell lymphoma cells (156). Directly in the context of TPD, however, evidence that PROTAC-mediated degradation itself engages compensatory autophagic flux remains limited, and whether tumors can bypass TPD-induced ubiquitination through compensatory autophagy therefore requires prospective evaluation in degrader-treated tumors (157). It should also be noted that autophagy-based degraders themselves face tissue-specificity and delivery challenges, and their utility in UPS-compromised tumors remains to be established (31–33). Moreover, target localization and accessibility—together with the expression patterns, localization and activity of E3 ligases, deubiquitinases and the wider ubiquitin machinery—are critical parameters in the exploitation of PROTACs (157), indicating that monitoring E3 ligase and DUB status together with autophagic flux should be part of resistance-prediction strategies (157). Because the resistance mechanisms described above converge on the ubiquitin-proteasome system, degradation routes that do not depend on proteasomal function represent mechanistically distinct options in UPS-compromised settings: for intracellular targets, autophagy-based modalities such as AUTACs, ATTECs and AUTOTACs engage the autophagy machinery rather than the proteasome (31–33); for membrane and extracellular targets, endolysosomal modalities such as LYTACs achieve degradation by trafficking proteins to the lysosome (7, 16). However, direct evidence of the activity of these routes specifically in UPS-compromised tumors is lacking, and their clinical maturity remains limited; rather than assuming that alternative degradation routes will be active in resistant tumors, resistance prediction should integrate E3 ligase, DUB and autophagic-flux monitoring (157) with rational combination strategies. Together, these considerations provide a balanced view of the long-term efficacy of TPD.

Beyond the design of the degrader molecule itself, delivery to the tumor site presents a third and equally formidable challenge. The clinical translation of TPD technologies for cancer therapy remains substantially constrained by the need for precise and efficient delivery to tumor tissues, although various delivery systems have been developed—including prodrugs, nanoparticles, and platforms based on proteins, nucleic acids, cells, or two--dimensional materials (158). However, delivery strategies tailored for TPD still face fundamental challenges. For instance, activation--based approaches designed for spatiotemporal control—such as photo--activated PROTACs, hypoxia--activated PROTACs, and trivalent PROTACs—are conceptually promising. Yet their clinical application is often limited by insufficient tissue penetration, restricted specificity for tumor hypoxia, and poor oral bioavailability due to molecular complexity (159). This reflects the persistent challenge of balancing control precision with practical applicability in current delivery systems. On the other hand, strategies employing biological carriers—such as degradable platelets (DePLTs)—which covalently anchor protein of interest (POI) ligands to heat shock protein 90 (HSP90) within platelets, can exploit the natural tropism of platelets toward sites of hemorrhage to achieve targeted protein degradation in postoperative regions (160). However, the efficacy of this strategy critically depends on the large--scale activation of platelets at hemorrhagic sites, posing a significant challenge for its application in most solid tumors lacking acute bleeding signals. While platelet infiltration exists within the tumor microenvironment, whether their activation state and extent are sufficient to effectively trigger the DePLT--mediated degradation program remains an open question. Thus, moving beyond the limitations of current delivery paradigms to develop a next--generation platform that is not constrained by specific pathological contexts and can universally target and enrich in diverse tumor tissues has become a pivotal scientific challenge for advancing the field (**Figure 6**).

PROTACs still face the challenges of low solubility, poor permeability, off-target effects and metabolic instability (161), which translate into four delivery barriers for solid tumors—tissue penetration, oral bioavailability, metabolic stability and tumor-specific delivery. Regarding tissue penetration, inadequate delivery of degraders to the target site is a significant hurdle to their *in vivo* application (158), and the dense extracellular matrix together with elevated interstitial fluid pressure within solid tumors establishes a transport-limiting system that restricts drug penetration (162). These physical barriers are being actively addressed: tumor-penetrating iRGD peptide-engineered PROTAC nanoparticles achieve deep tumor penetration via neuropilin-1-mediated transcytosis (163), ginsenoside Rh2-functionalized liposomes enhance PROTAC delivery through ECM-remodeling collagen degradation (164), and nanoparticle-based delivery systems are being developed to improve tissue distribution and target-site localization (165). Together with the size of nanocarrier-based degraders, these physical barriers make deep penetration the dominant bottleneck for solid tumors, and combinatorial approaches that couple penetration enhancers with tumor-homing ligands are being explored (165). Regarding oral bioavailability, PROTACs occupy the beyond-Rule-of-Five chemical space, with molecular weights often exceeding 700 Da (29), and reliable design of orally bioavailable PROTACs remains challenging (166): dedicated optimization—for example, restricting solvent-exposed hydrogen-bond donors and controlling conformational chameleonicity (167), together with strategies such as intramolecular hydrogen bonding, macrocyclization, dosage and formulation that can improve the bioavailability of beyond-Rule-of-Five oral drugs (168)—is required, and an empirically preferred oral-PROTAC property space proposed by one research group (no more than 3 hydrogen-bond donors, molecular weight no more than 950 Da, and no more than 12 rotatable bonds) has been suggested to guide design (169), and reducing the Caco-2 efflux ratio has emerged as a useful addition to these design guidelines (170). Despite these hurdles, orally administered degraders such as ARV-110, ARV-471 and BMS-986365 have demonstrated measurable systemic exposure and pharmacodynamic activity in clinical studies (166). Regarding metabolic stability, the metabolism of PROTACs cannot be predicted from that of their constituent ligands, and the chemical nature and length of the linker play a major role in metabolic liability (42); because an ideal linker should be stable in the circulatory system and release its payload specifically in the tumor (43), linker design is a key determinant of degrader metabolic stability, and tailored *in vitro* DMPK assay cascades using experimentally determined values have been proposed to improve the translation of clearance predictions for degraders (169). Regarding tumor-specific delivery, three complementary strategies are emerging: activation-based prodrugs—for example, PROTAC prodrugs whose azo-based linker is selectively cleaved in the hypoxic tumor microenvironment, releasing the active degrader and mitigating off-target systemic toxicity, with photodynamic stimulation further promoting activation (171); biological carriers such as degradable platelets (DePLTs) that target wound areas and undergo activation for local degradation (160); and self-assembling or pretargeted peptide platforms that concentrate the degrader at the tumor site before activation (172). All three delivery strategies described above remain at the preclinical stage, and their clinical maturity is not yet comparable to that of the orally bioavailable degraders discussed above. Nevertheless, tissue penetration remains the least solved of the four barriers, and the clinical translation of these delivery strategies has yet to be demonstrated. Together, deep-tumor penetration, oral bioavailability, metabolic stability and tumor-selective activation constitute the key design criteria for next-generation degraders, and combining compact bRo5 scaffolds with environment-responsive delivery will likely be required for clinical success.

Compared with traditional oral small-molecule drugs, whose molecular weights are typically below 500 Da, PROTACs occupy a distinct beyond-Rule-of-Five (bRo5) chemical space, with molecular weights often exceeding 700 Da (3, 29). Analyses of marketed oral drugs and clinical candidates show that oral drugs can be found far beyond the rule of 5, and that properties such as intramolecular hydrogen bonding, macrocyclization, dosage and formulations can be used to improve bRo5 bioavailability (168); interrogating this space requires advanced tools such as the experimental measure of exposed polarity (EPSA) and the AbbVie multiparametric score (AB-MPS), and a high-throughput polarity-reduction descriptor (ETR, the ratio of EPSA to topological polar surface area) highlights unique bRo5 and PROTAC subsets that warrant specialized drug-design strategies for effective absorption (167). The bifunctional architecture of PROTACs, which is composed of a target-protein ligand, an E3 ligase ligand and a linker (29), is central to this constraint: the large size and complex structure of PROTACs can reduce their stability and cellular permeability, and their high molecular weight has been linked to poor solubility and cell permeability (29). The magnitude of this deficit is illustrated quantitatively by the KT-474 discovery campaign, in which the benchmark IRAK4 degrader exhibited modest passive permeability (Papp = 4.8 × 10^-^^6^ cm/s) and a high MDR1 efflux ratio (ER = 35), with no oral absorption in rats, whereas the conventional immunomodulatory small molecule pomalidomide displayed a markedly higher Papp of 25.4 × 10^-^^6^ cm/s (137). Even after extensive structure-based optimization, the resulting clinical candidate achieved oral bioavailabilities of only 12%, 35% and 13% in rats, dogs and monkeys, respectively (137), and vepdegestrant (ARV-471)—the most clinically advanced ER-targeting PROTAC (29)—similarly displayed low-to-moderate oral bioavailability (17.9% in mice and 24.1% in rats), with incomplete absorption attributed to its low solubility and permeability owing to its high molecular weight (173). These values contrast with the rule-of-five paradigm, which was established to prioritize compounds with an increased likelihood of high oral absorption (168). At the molecular level, experimental and computational studies show that the propensity of PROTACs to adopt folded conformations with a low solvent-accessible 3D polar surface area in an apolar environment is correlated with high cell permeability (174), and molecular dynamics simulations may be used for the prospective ranking of cell permeability in the design of cereblon PROTACs (174). Accordingly, multiparameter scoring such as AB-MPS has been applied to large PROTAC datasets, revealing new patterns of physicochemical trends that inform design (167). A key design principle emerging from structure-guided optimization is restricting solvent-exposed hydrogen-bond donors, which improved passive permeability in the KT-474 campaign (137). Zhang et al. used dimensionality reduction analysis and model molecule validation to identify key structural features for improving the oral absorption of BTK-PROTACs, leading to compound C13 with improved oral bioavailability, high degradation activity and selectivity (175). The PROTAC field has generated numerous studies that expand our understanding of the unique mode of action and advantages of PROTACs, spurring interest in PROTACs as a novel therapeutic strategy (176). These frameworks provide a practical toolbox for bRo5 PROTAC optimization (137, 167, 168, 174, 175).

Monitoring the pharmacodynamic effects of degraders in early-phase trials is complicated by the difficulty of sampling tumor tissue, particularly in inaccessible sites. Liquid biopsy—most prominently circulating tumor DNA (ctDNA)—has demonstrated translational potential for prognostication, molecular profiling and monitoring (177), and can inform TPD trials in three ways. First, serial ctDNA measurements can serve as a surrogate readout of disease response: in oncogene-driven advanced NSCLC treated with tyrosine kinase inhibitors, ctDNA clearance within 10 weeks of treatment initiation was associated with improved overall and progression-free survival (178), and early on-treatment clearance of KRASG12C ctDNA refined clinical response prediction in sotorasib-treated patients (179). Second, targeted ctDNA panels can detect emerging resistance alterations at the genomic level—for example, alterations in KRAS, EGFR, BRAF and MAP2K1 that bypass KRAS G12C inhibition and reduce therapy efficacy (180). Third, because ctDNA reflects tumor burden and genomic changes rather than target-protein depletion itself, it must be complemented by direct pharmacodynamic biomarkers—exemplified by the >80% degradation of BTK measured in the blood of patients receiving the degrader NX-2127 (181)—and, whenever feasible, by tissue biopsies. Integrating ctDNA kinetics into phase I/II dose-finding designs is therefore feasible (182), although direct quantification of target degradation remains the definitive pharmacodynamic readout for TPD.

## 6 Outlook

The clinical ceiling of current targeted protein degradation is not set by molecular potency but by biological selectivity. Most degraders recruit ubiquitously expressed E3 ligases such as CRBN or VHL, making on-target, off-tissue toxicity the dose-limiting barrier rather than pharmacological efficacy. While proof-of-concept platforms like SelecTAC exploit lineage-restricted receptors to confine degradation to specific immune subsets, solid tumors rarely offer such discrete surface markers (183). The key engineering priority, therefore, is to identify E3 ligases or endocytic receptors with expression tightly restricted to the tumor microenvironment or specific stromal compartments, creating an AND-gate logic in which productive degradation requires simultaneous engagement of both a tumor-associated antigen and the target protein. Beyond hunting for rare receptors, the field must also develop strategies decoupled from specific receptor overexpression altogether—degraders that respond to universal physicochemical hallmarks of malignancy, such as acidic pH, elevated redox potential, or dysregulated protease activity—thereby ensuring robust activity across genetically heterogeneous tumors without relying on a single, often unstable, biomarker.

Degrader efficacy *in vivo* is conditioned by the cellular context of the target—shaped by dynamic regulatory layers such as long non-coding RNAs and epitranscriptomic modifications (184, 185), assessable by in-cell structural methods such as NMR (186, 187), and influenced by the context-dependent activity of small-molecule modulators (188, 189)—underscoring that TPD design must extend beyond ligand chemistry to the disease-associated networks in which targets operate.

This AND-gate architecture means that their degradative function strictly depends on the synchronous engagement of a gating receptor and the target protein, which together form a stable ternary endocytic complex that triggers lysosomal internalization and degradation (183). Because a cell expressing only the gating receptor or only the target protein cannot form this functional complex, degradation occurs selectively in cells co-expressing both molecules (183, 190). In the prototype design, CD22 serves as the B-cell-specific lysosome-targeting receptor, restricting degradation to B cells (183). However, this design, which relies on a receptor largely restricted to the B-cell lineage, cannot be directly generalized to solid tumors, which lack strictly lineage-restricted surface markers. To address this bottleneck in future designs, a dual-tumor-associated-antigen cooperative recognition mode—in which the degradation program would be initiated only in tumor cells bearing a specific antigen combination—has been proposed as a conceptual upgrade of the SelecTAC logic, drawing design inspiration from AND-gate signaling concepts established in cellular engineering (190). At the molecular level, the feasibility of implementing “AND” logic-gated activation in targeted degradation has been illustrated by a Split-Deliver-Click nanoplatform, in which split PROTAC precursors are activated and released only upon co-triggering by tumor-overexpressed enzymes legumain and cathepsin B (191); although this system relies on intracellular enzyme-triggered activation rather than cell-membrane dual-antigen recognition, it demonstrates that AND-gate activation logic can be implemented in PROTAC-based degradation. Such dual-constraint strategies could substantially enhance biological selectivity and circumvent the target-dependent off-tissue toxicity that is pervasive in solid-tumor TPD (190). The bispecific T-cell engager (BiTE) field has accumulated extensive clinical experience in broadening the indications of T cell-engaging therapies toward common solid cancers, and these design concepts could inform analogous strategies in targeted protein degradation (192). The future application of SelecTAC platforms in solid tumors will depend on the realization of such dual-antigen gating strategies; inter-patient and intratumoral heterogeneity in the co-expression profiles of dual targets, as well as additional molecular design complexity, remain key challenges.

Yet mastering protein destruction addresses only half of the therapeutic equation. Tumors frequently co-opt deubiquitinating enzymesto stabilize pro-oncogenic and immunosuppressive proteins, extending their half-lives beyond normal limits. Engineering the converse modality—molecular stabilizers that selectively shield immunostimulatory factors from proteolytic clearance—offers a compelling complementary strategy. Platforms such as deubiquitinase-targeting chimeras or MGs that enhance the stability of key immune activators could function as the yang to PROTACs’ yin, transforming targeted protein degradation from a unidirectional elimination toolkit into a bidirectional proteostasis rheostat capable of both silencing suppressive nodes and amplifying effector signals.

Ultimately, the maturation of TPD in immuno-oncology will depend less on expanding the target roster than on integrating these advances into a programmable system for protein-fate control. By combining tissue-selective delivery vectors, environment-responsive activation switches, and bidirectional stabilization–degradation modules, the field can move beyond static elimination toward dynamic, reversible reprogramming of the tumor immune microenvironment.
